# Epstein‐Barr Virus Infection at Single‐Cell Resolution

**DOI:** 10.1002/jmv.70825

**Published:** 2026-02-05

**Authors:** Elliott D. SoRelle

**Affiliations:** ^1^ Department of Microbiology & Immunology University of Michigan Ann Arbor Michigan USA; ^2^ Department of Biological Chemistry University of Michigan Ann Arbor Michigan USA

**Keywords:** autoimmunity, B cell lymphoma, EBV, infectious mononucleosis, multiple sclerosis, nasopharyngeal carcinoma, NK/T cell lymphoma, scATAC‐seq, scRNA‐seq, spatial‐omics

## Abstract

Epstein‐Barr virus (EBV) infection has been studied at single‐cell resolution for six decades and counting. Such investigations can reveal virus‐host interactions and their dependence on viral strain, cellular niche, infection program, immune response regulation, and time. Understanding these factors is paramount to treating EBV‐associated cancers and autoimmune diseases. This review examines the state of the field in EBV single‐cell and spatial‐omics spanning experimental models and clinical samples. Topics of primary interest include the growing adoption and emerging biological themes from single‐cell assays and analyses, the shift from characterization toward functional single‐cell studies, and strategies to maximize clinically relevant insights from dense single‐cell and spatial datasets. Ancillary topics include the historical evolution of the single‐cell EBV field and end‐to‐end single‐cell sequencing workflows. Special attention is given to open questions in molecular mechanisms of EBV pathogenesis and how they might be resolved by future studies utilizing single‐cell techniques.

**Abbreviations**
ABC: atypical B cell
AITL: Angioimmunoblastic T cell lymphoma
Akata: Burkitt Lymphoma‐derived cell line
ALI: Air‐liquid interface
AP: activated precursor (B cell state)
ATAC‐seq: assay for transposase‐accessible chromatin sequencing
BCR: B cell receptor
BL: Burkitt Lymphoma
CAEBV: chronic active EBV
cDNA: complementary DNA
ChIA‐PET: chromatin interaction analysis with paired‐end tagging
cHL: classic Hodgkin lymphoma
CITE‐seq: cellular indexing of transcriptomes and epitopes sequencing
cLN: cervical lymph node
CNV: copy number variation
CODEX: co‐detection by indexing
CosMx: NanoString platform for spatial imaging transcriptomics
CSC: cancer stem cell
CSF: cerebrospinal fluid
DE: differential expression/differentially‐expressed (as in genes)
DLBCL: diffuse large B cell lymphoma
DN2: double‐negative type 2 cells (IgD‐CD27‐ B cell population)
drop‐seq: droplet‐based single‐cell library preparation technique
EBER: EBV‐encoded small RNAs
EBER‐iSH: EBER in situ hybridization
EBNA: EBV nuclear antigen (viral latency genes)
EBV: Epstein‐Barr virus (HHV‐4)
EBVSE: EBV super enhancer (gene regulatory complex)
ELISA: Enzyme‐linked immunosorbent assay
FACS: fluorescence‐activated cell sorting
flow‐FiSH: flow cytometry fluorescence in situ hybridization
GA: gastric adenocarcinoma
GC: germinal center
GRN: gene regulatory network
HHV: human herpesvirus
HIV: human immunodeficiency virus
HIV‐NHL: HIV‐associated non‐Hodgkin lymphoma
Hi‐C: chromatin conformation capture technique
HL: Hodgkin lymphoma
HLA: human leukocyte antigen
HLH: hemophagocytic lymphohistiocytosis
HRS: Hodgkin Reed‐Sternberg (HL malignant cell)
HSC: hematopoietic stem cells
HSCT: hematopoietic stem cell transplantation
IF: immunofluorescence
IM: infectious mononucleosis
IRF: interferon regulatory factor
ISH: in situ hybridization
K‐NN: k‐nearest neighbors
LCL: lymphoblastoid cell line
LMP: latent membrane protein (viral latency genes)
MHC: major histocompatibility complex
MIS‐C: multisystem inflammatory syndrome in children (SARS‐CoV‐2 sequelae)
mRNA: messenger RNA
MS: multiple sclerosis
NALT: nasopharynx‐associated lymphoid tissue (murine equivalent to tonsillar tissue)
NGS: next‐generation sequencing
NHL: non‐Hodgkin lymphoma
NKTCL: NK/T cell lymphoma
NK cell: natural killer cell
NPC: nasopharyngeal carcinoma
NPC43: nasopharyngeal carcinoma cell line
ORF: open reading frame
PBMC: peripheral blood mononuclear cells
PC: principal component
PCA: principal component analysis
PCR: Polymerase chain reaction
PLWH: people living with HIV
PSC: primary sclerosing cholangitis
pSS: primary Sjögren's syndrome
PTLD: Post‐transplant lymphoproliferative disorder
PWMS: people with multiple sclerosis
QC: quality control
RA: rheumatoid arthritis
RNA‐seq: RNA sequencing
scATAC‐seq: single‐cell assay for transposase‐accessible chromatin sequencing
scChIP‐seq: single‐cell chromatin immunoprecipitation sequencing
scMNase‐seq: single‐cell micrococcal nuclease sequencing
scRNA‐seq: single‐cell RNA sequencing
scVDJ‐seq: single‐cell variable, diversity, joining segment sequencing (general technique for scBCR/TCR‐seq)
SLE: systemic lupus erythematosus
split‐seq: Plate‐based single‐cell barcoding library preparation strategy
TCR: T cell receptor
TGF‐β: transforming growth factor beta
TME: tumor microenvironment
TR: tandem repeat
t‐SNE: t‐distributed stochastic neighbor embedding
UMAP: uniform manifold approximation projection
UMI: unique molecular identifier
Visium HD/V2: 10× Genomics spatial transcriptomics platform
VRC: Viral replication compartment

## 1 Main

1.1

“That gum you like is going to come back in style.” [1]

The first remarkable electron micrograph from Epstein, Barr, and Achong's landmark 1964 report on lymphoblasts derived from Burkitt Lymphoma (BL) is the earliest direct evidence of Epstein‐Barr virus (EBV; human herpesvirus 4) [2]. Beyond foundational importance to human tumor virology, this initial rendering of the virus captured core dynamics of virion maturation within an individual cell. Epstein and colleagues contextualized this observation with an intriguing detail – only “one in several score” of cells exhibited viral particles [2]. The authors conceded possible inaccuracy due to sample preparation, but the infrequency of cells harboring active virion production (lytic replication) would be confirmed many times over as hallmark latent infection in B cells [3]. The discovery of EBV thus doubled as the first evidence of the virus's dynamics and inherent biological heterogeneity.

**Figure 1:**
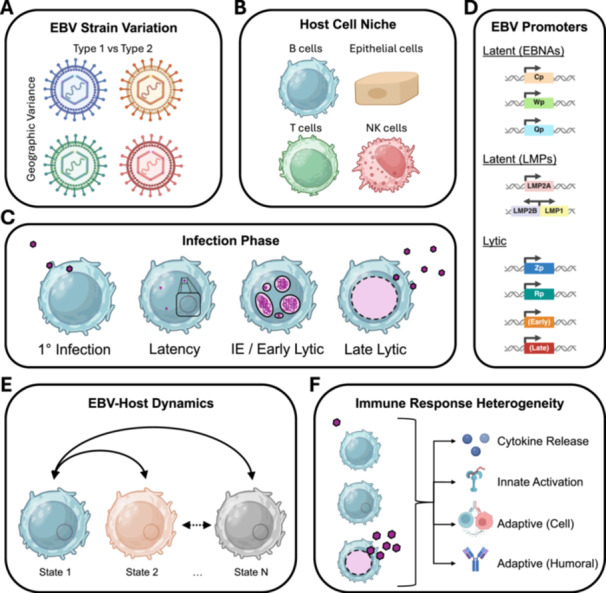
Viral and host sources of single‐cell heterogeneity in EBV‐infected samples. (A) EBV strains are classified into two types. EBV type and other viral genetic aspects vary with geography. (B) EBV can infect diverse human cell types including lymphocytes (B, T, and NK cells) and epithelial cells (nasopharyngeal, gastric, and other). (C) EBV infection occurs in multiple distinct phases. Characteristic infection phase varies by host cell niche. (D) Latent and lytic infection exhibit additional heterogeneity based on combinatorial activity from multiple promoters within the EBV genome. (E) EBV‐infected cells occupy dynamic states driven by host‐virus interactions. (F) Immune responses from EBV‐infected cells (e.g., cytokine release) and responses to infected cells (innate and adaptive) vary by viral program. *(Figure created with BioRender under license)*.

Sixty years on, key sources of EBV infection complexity have come into sharper focus (Figure 1). Consequently, so too has the need for high‐resolution experimental and analytic methods. The range of available single‐cell techniques was limited in the early decades following the discovery of EBV (Supplementary Text, Section IA, “Early Single‐Cell Insights”). Nonetheless, classical methods successfully defined fundamental EBV infection aspects underlying viral malignancies. The field leveraged single‐cell and pseudobulk experimental innovations, especially advances in fluorescence microscopy and flow cytometry, through the 1980s, 1990s, and early 2000s (Supplementary Text, Section IB, “Advances in Single‐Cell EBV Methods”). These techniques helped refine conceptual models of primary, latent, and lytic phase dynamics; identify memory B cells as the classic in vivo reservoir for latent EBV [4]; and reveal clinically relevant viral heterogeneity and drug responses. From the 2010s to the present, EBV researchers adopted genome‐wide approaches (pseudobulk, single‐cell, and spatial‐omics) to investigate experimental models and clinical specimens from virus‐associated cancers and autoimmune disorders (Supplementary Text, ibid.). The single‐cell next‐generation sequencing (NGS) era has borne new understanding of EBV‐associated diseases in considerable molecular detail.

**Figure 2:**
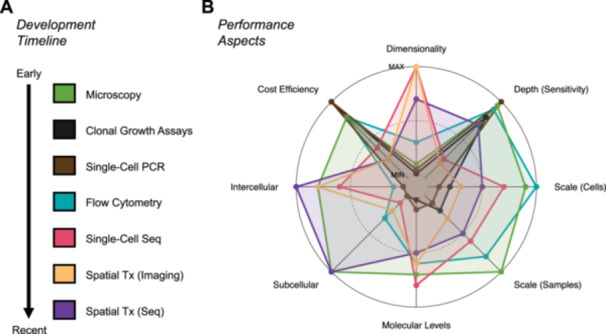
Evolution and relative performance of single‐cell assays. (A) Single‐cell experimental techniques chronologically ordered by first use in the EBV field. (B) Spider plot depicting qualitative comparison of single‐cell technique performance. Points are color‐coded by technique. Increasing radial distance denotes better performance in a given aspect.

Though methods to assay individual cells are far from new, recent enhancements in the molecular breadth, depth, sensitivity, and scale of single‐cell approaches constitute a profound technical shift (Figure 2). These capabilities have yielded a robust body of high‐resolution characterization datasets from experimental models and clinical biosamples as well as an emerging trend toward functional genetic and pharmacologic single‐cell applications. Beyond experimental designs and technologies lies an ever‐growing arsenal of bioinformatic tools to discover and quantify viral phenotypes, biological functions, dynamics, and cell interactions – and to build integrated phenotypes across molecular modalities. Consequently, it is critical to mitigate practical barriers to single‐cell experimentation and increase the accessible use of single‐cell analyses for future research. While these powerful technologies have characterized previously unknown facets of EBV‐associated diseases, resolving long‐standing questions in the molecular mechanisms of EBV pathogenesis ultimately depends on applying the appropriate high‐resolution methods in carefully designed studies.

## 2 Single‐Cell and Spatial Sequencing (ca. 2020–Present): New Dimensions of EBV Infection

**Figure 3:**
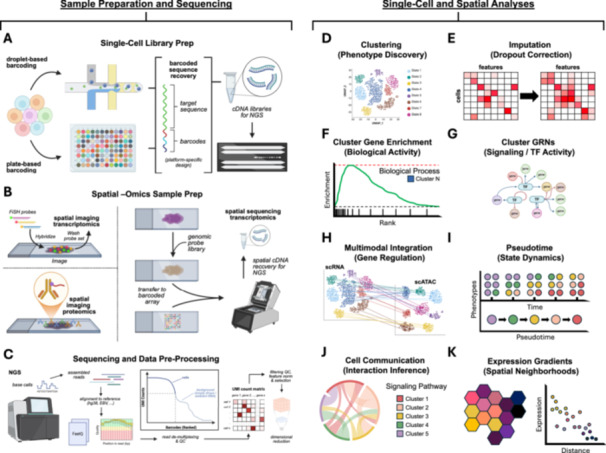
Select single‐cell and spatial analyses. (A) Schematic of droplet‐ and plate‐based single‐cell library preparation strategies. (B) Schematic of image‐based spatial assays (transcriptomics and proteomics; left side) and genome‐wide spatial sequencing library preparation (right side). (C) Overview of next‐generation sequencing (NGS), read assembly, reference genome alignment, quality control (QC), generation of unique molecular identifier (UMI) count matrices, and dimensional reduction. (D) Single‐cell clustering to discover biological phenotypes. (E) Optional correction of transcript dropout (matrix sparsity) with imputation methods. (F) Gene enrichment and ontology analyses of cluster‐specific differentially expressed (DE) genes. (G) Application of gene regulatory network (GRN) analyses to infer cluster‐specific signaling pathways and transcription factor (TF) activity from RNA expression. (H) Anchor‐based integration of single‐cell data collected across different molecular levels/technologies. (I) Deconvolution of mixed cell biological states in timepoint data into pseudotime‐ordered state trajectories. (J) Inference of cell‐cell ligand‐receptor interactions from cluster‐specific expression. (K) Incorporation of spatial information to define expression gradients relative to tissue or cell types of interest. *(Figure created with BioRender under license)*.

Single‐cell and spatial‐omics techniques have revolutionized experimental and clinical biological research. A comprehensive review of these methods falls outside the scope of this review but can be found elsewhere [5, 6]. Generally, single‐cell sequencing relies on combining three elements: barcoded molecular capture of sequence features in cells, high‐throughput sequencing of the barcoded features, and per cell feature quantification via barcode‐demultiplexed mapping to a reference genome. Spatial sequencing is highly analogous, with the exceptions that samples are typically intact tissues and molecular capture incorporates spatially arrayed barcodes (which define assay resolution) for *in situ* expression reconstruction. Single‐cell and spatial techniques can measure one or more molecular layers (modalities). These include gene expression (scRNA‐seq) [7, 8, 9, 10], RNA plus surface protein expression (CITE‐seq) [11], accessible chromatin (scATAC‐seq; scMNase‐seq for additional nucleosome positioning) [12, 13], immune receptor profiling (scVDJ‐seq) [14], chromatin factor footprinting (scChIP‐seq) [15], and genome‐wide chromatin conformation (single‐cell Hi‐C) [16]. Many of these assays involve variations on a core workflow of single‐cell sample preparation, high‐throughput sequencing, and downstream computational and informatic analyses (Figure 3). A detailed walkthrough of a general single‐cell sequencing workflow for gene expression (scRNA‐seq) is provided as a supplement for interested readers (Supplementary Text, Section II).

The core technical advantage of single‐cell and spatial sequencing resides in measurements acquired at high biological resolution across a broad feature space. Together, these aspects enable low‐bias discovery and quantification of high‐dimensional cell phenotypes within complex samples. Such experimental methods are especially valuable for studying viral infections, which often compound intrinsic cellular heterogeneity. Thus, clusters (pseudobulk phenotypes) delineated from single‐cell or spatial sequencing of EBV^+^ samples should ideally reflect their constituent infected and uninfected cells. Once accurately defined, clusters form the basis for measuring cell state frequencies, discerning their respective biological programming, inferring infection dynamics, and predicting cell‐cell interaction axes across EBV^+^ cells, immune cells, and other bystander cell types in a given sample. Spatial sequencing allows these putative interactions to be assayed *in situ*. A list of EBV‐related single‐cell and spatial‐omics studies and datasets is provided herein (Table 1). The following sections aim to distill specific insights and key biological themes from these studies. Sections are organized by experimental models versus clinical samples and cell/tissue type for clarity.

**Table 1:** Single‐cell and spatial studies of EBV infection models and associated diseases.

Single‐cell sequencing					
Experimental models					
Cell type	EBV infection model	Assay/technology	Study	Data access	Notes
B cell	LCL	scRNA‐seq/10x Genomics single‐cell RNA 3' v2	Ref. [18]	SRA: SRP172838; GEO: GSE126321	LCL scRNA‐seq (no EBV gene alignment)
B cell	LCL	scRNA‐seq/10x Genomics single‐cell RNA 3' v2	Ref. [19]	SRA: SRP284226; GEO: GSE158275	LCL transcriptomic continuum from early activation to plasmablast differentiation associated with viral latency program
B cell	LCL	scRNA‐seq/10x Genomics single‐cell RNA 3' v2	Ref. [23]	SRA: SRP284226; GEO: GSE158275	T‐bet^+^ CXCR3^+^ B cell subsets in LCLs
B cell	LCL	scRNA‐seq/10x Genomics single‐cell RNA 3'	Ref. [24]	SRA: SRP354696	EBV strain type‐dependent cellular gene expression and lytic reactivation propensity
B cell	LCL (with HHV‐6)	scRNA‐seq/10x Genomics single‐cell RNA 3' v3.1	Ref. [26]	SRA: SRP433697; GEO: GSE230239	LCLs from individuals with chromosomally integrated human herpesvirus 6 A/6B (HHV‐6)
B cell	1° infection in PBMCs; disaggregated tonsils	scRNA‐seq + scATAC‐seq/10x Genomics single‐cell Multiome; 10x Genomics single‐cell RNA 3' v3.1	Ref. [27, 28]	SRA: SRP346796; GEO: GSE189141; SRA: SRP287808; GEO: GSE159674	Time‐resolved multiomics of peripheral blood B cell primary infection and comparison to tonsillar B cell scRNA‐seq phenotypes
B cell	BL lines (P3HR1‐ZHT, Akata); B95‐8‐ZHT	scRNA‐seq/10x Genomics single‐cell RNA 3' v3.1	Ref. [38]	SRA: SRP521357; GEO: GSE272763	Time‐resolved lytic reactivation in cell lines; distinct abortive and full lytic response states
B cell	1° infection; EBNA‐LP KO	scRNA‐seq/10x Genomics single‐cell RNA 3' v4	Ref. [37]	SRA: SRP546381; GEO: GSE282400	Identification of SP140L as 1° infection restriction factor from time‐resolved scRNA‐seq with EBNA‐LP KO recombinant virus
B cell	1° infection transwell migration assay	scRNA‐seq/SMART‐seq. 2 single‐cell RNA	Ref. [36]	SRA: SRP583147	Comparison of migrating and non‐migrating B cells upon 1° infection; EBV induces FAK‐dependent diapedesis
Humanized mouse; B cells from spleen and brain	in vivo 1° infection	scRNA‐seq + scVDJ‐seq/10x Genomics single‐cell RNA universal 5' v2 + V(D)J enrichment	Ref. [35]	SRA: SRP592606; GEO: GSE299939; Zenodo: doi. org/10.5281/zenodo.15602185	1° infection induces migration of EBV^+^ T‐bet^+^ CXCR3^+^ B cells to the central nervous system (CNS) in humanized mice; CNS‐resident EBV^+^ B cells attract EBV‐specific CD8^+^ effector memory T cells and CD4^+^ Th17 cells
Humanized mouse; NALT & spleen T effector memory cells	in vivo 1° infection	scRNA‐seq/10x Genomics single‐cell RNA 3' v3.1	Ref. [34]	ArrayExpress: E‐MTAB‐13853	1° infection induces tissue‐resident memory T cells (T_RM_) in mucosal tissues including mouse NALT (tonsil equivalent); infection/viral load control by systemic CD8^+^ effector memory T cells rather than tissue‐resident T_RM_
Epithelial cell	Nasopharynx‐derived pseudo air‐liquid interface culture	scRNA‐seq/10x Genomics single‐cell RNA 3' v3.1	Ref. [45]	SRA: SRP279565; GEO: GSE157243	Lytic infection in suprabasal epithelial cells and latent infection in basal, mucosecretory, and ciliated cells *in vitro*; correlated expression with NPC cell types
Epithelial cell	NPC lines	scRNA‐seq/10x Genomics single‐cell RNA 3' v3.1	Ref. [46]	SRA: SRP505924; GEO: GSE266679	Characterization of EBV^+^ NPC43; cells resistant to chemically‐induced lytic infection exhibit elevated SOX2 and NTRK2 (no EBV gene alignment)

Clinical samples: Cancer/Lymphoproliferative Disease					
Tumor cell of origin	Disease	Assay(s)	Study	Data Access	Notes
B cell	Hodgkin Lymphoma (HL)	scRNA‐seq/10x Genomics single‐cell RNA 3' v2	Ref. [48, 88]	EGA: EGAS00001005541	22 HL samples (5 EBV^+^); increased to 28 samples in subsequent study; Immunosuppressive LAG3^+^ T cell enrichment independent of EBV status
B cell	Diffuse large B cell lymphoma (DLBCL)	scRNA‐seq/10x Genomics single‐cell RNA 3' v2	Ref. [49]	CNGBdb: CNP0001940	17 DLBCL (2 EBV^+^); EBV^+^ samples include one ABC‐DLBCL and one GCB‐DLBCL
B cell	HIV‐associated BL	scRNA‐seq/10x Genomics single‐cell universal 5'	Ref. [50]		Evidence of heterogeneous latency programs in tumor cells from two EBV^+^ BL
B cell	PBMCs from pediatric PTLD	scRNA‐seq/10x Genomics single‐cell RNA 3' v3.1	Ref. [51]		Immune exhaustion signatures in PBMCs from two samples of EBV^+^ PTLD
NK/T cell	NKTCL	scRNA‐seq/10x Genomics single‐cell RNA universal 5'	Ref. [52]	GEO: GSE203663	10 EBV^+^ NKTCL and patient‐matched PBMCs; LMP1 association with PD‐L1 in immunosuppressive NK cell state
NK/T cell	NKTCL	scRNA‐seq/10x Genomics single‐cell RNA 3' v3.1	Ref. [53]	Available upon request	Incorporation of EBER capture probes for improved viral detection sensitivity
Epithelial cell	NPC	scRNA‐seq/10x Genomics single‐cell RNA 3' v2	Ref. [54]	CNGBdb: CNP0000428; GEO: GSE150430	15 NPC samples (13 EBV^+^); tumor immune response signatures associated with patient survival
Epithelial cell	NPC	scRNA‐seq/SMART‐seq. 2 single‐cell RNA & 10x Genomics single‐cell RNA 3' v2	Ref. [55]	BIG GSA: HRA000087	19 EBV^+^ NPC and 7 non‐malignant biopsies; inhibitory receptor expression in tumor‐infiltrating CD8^+^ T cell subsets
Epithelial cell	NPC	scRNA‐seq/10x Genomics single‐cell RNA 3' v2	Ref. [56]		Intratumoral phenotypic heterogeneity and oligoclonal intratumoral T cells; upregulated LAG3 expression in T cells
Epithelial cell	NPC	scRNA‐seq + scVDJ‐seq/10x Genomics single‐cell RNA universal 5' + V(D)J enrichment	Ref. [57]	SRA: SRP262300; GEO: GSE150825	14 NPC samples; T cell subset exhaustion (TIM3/HAVCR2) and immunoregulatory (FOXP3) signatures in tumors; clonal expansion of tumor‐infiltrating B cells; immunosuppressive myeloid signature; microenvironmental immune signatures correlation with disease prognosis
Epithelial cell	NPC & matched PBMCs	scRNA‐seq + scVDJ‐seq/10x Genomics single‐cell RNA universal 5' + V(D)J enrichment	Ref. [58]	BIG GSA: HRA000159; GEO: GSE162025	10 NPC‐blood sample pairs; peripherally‐derived exhausted CD8^+^ (CTL) and TNFRSF4^+^ (T_reg_) enrichment in tumors; immunosuppressive axis along these T cells, malignant cells, and LAMP3^+^ dendritic cells
Epithelial cell	NPC	scRNA‐seq + imaging MC/10x Genomics single‐cell RNA 3' v3	Ref. [89]	BIG GSA: HRA003340	7 NPC samples; MHC downregulation in tumor cells; copy number variation among malignant cells; evidence of non‐malignant epithelial cell mediation of immunosuppressive environment; CD8^+^ NK cell subset
Epithelial cell	Gastric carcinoma	scRNA‐seq/10x Genomics single‐cell RNA 3' v2	Ref. [60]	BIG GSA: HRA000051; available upon request	9 gastric carcinoma samples (one EBV^+^); upregulated lymphocyte activation and immune response signatures in EBV‐specific cell tumor cluster; MHC class II upregulation; phenotypic skewing toward immune expression
Epithelial cell	Gastric carcinoma	scRNA‐seq + scVDJ‐seq/10x Genomics single‐cell RNA universal 5' + V(D)J enrichment	Ref. [61]		6 gastric carcinoma samples (3 EBV^+^); increased B and T cell infiltration of EBV^+^ GC tumors; oligoclonal lymphocytes with immune suppressive phenotypes; cytotoxic T cell subset expansion following PD‐1 immunotherapy

Clinical samples: Autoimmune/inflammatory disease					
Cell type/tissue	Disease	Assay(s)	Study	Data access	Notes
PBMCs	MS	scVDJ‐seq/in‐house plate‐based universal 5'	Ref. [33]	SRA: SRP346413	Identified antibody cross‐reactivity between EBNA1 and GlialCAM in people with MS
PBMCs	Early MS/clinically isolated syndrome (CIS)	scRNA‐seq + CITE‐seq/10x Genomics single‐cell RNA 5' v1 + ADT	Ref. [62]	SRA: SRP508353; GEO: GSE267750	32 early MS (CIS) samples from 16 individuals at two timepoints; TBX21^+^ CXCR3^+^ CD21^lo^ atypical B cell (ABC) phenotype enriched in early MS; EBV‐associated inflammatory and neurotropic expression signature in ABCs; EBV‐associated ABC signature significantly linked to longitudinal disease activity; viral reads not directly detected
Cervical lymph nodes	MS	scRNA‐seq + scVDJ‐seq + CITE‐seq/10x Genomics single‐cell RNA 5' v2 + V(D)J enrichment + ADT	Ref. [63]	Zenodo: 10.5281/zenodo.10006019	Double‐negative memory B cell subset (DN2) exhibiting host biomarkers of lytic EBV infection enriched in people with MS; increased frequencies of EBV‐specific CD8^+^ T cells; correlation with increased EBV viral loads and genome detection in draining cervical lymph nodes
PBMCs	Primary Sjogren Syndrome (PSS)	scRNA‐seq/10x Genomics single‐cell RNA 5'	Ref. [65]	BIG GSA: HRA001355	Indirect signatures of viral immune subset dysregulation (no EBV gene alignment)
PBMCs	Systemic Lupus Erythematosus (SLE)	scRNA‐seq + scVDJ‐seq/10x Genomics single‐cell RNA 5' + V(D)J enrichment	Ref. [66]	Available upon request	6 SLE samples (2 EBV^+^); increased frequency of proliferative CD8^+^ T cells and altered differentiation of CD4^+^ regulatory T cells (T_reg_, Th17) in SLE with detected EBV; reduced BCR clonality in EBV^+^ SLE; increased IgG1 and IgA vs IgM in EBV^+^ SLE
PBMCs	Pediatric Hemophagocytic Lymphohistiocytosis (HLH); Infectious Mononucleosis (IM)	scRNA‐seq/10x Genomics single‐cell RNA 3' v3.1	Ref. [71]	Japan SRA: DRA017005	3 pediatric HLH samples; 2 IM samples (all EBV^+^); impaired CD8^+^ T cell responses in HLH vs IM; upregulated LAG3^+^ T cells in EBV^+^ HLH with underlying chronic active EBV (CAEBV); T cell subset‐specific upregulation of interferon response
PBMCs	Chronic Active EBV (CAEBV)	scRNA‐seq/Singleron FocuSCOPE single‐cell RNA + EBV detection panel	Ref. [72]	GEO: GSE231946	6 CAEBV samples (including cases with HLH or LPD); EBV detection across lymphoid and myeloid cell types in CAEBV, including evidence in hematopoietic stem cells (HSCs)
PBMCs	Pediatric HLH & IM	scRNA‐seq/Singleron FocuSCOPE single‐cell RNA	Ref. [73]	BIG GSA: HRA01943	17 HLH & 9 IM samples; T cell NF‐κB activation signature enrichment versus healthy controls; enhanced inflammatory cytokine signatures in NK cells and monocytes from HLH and IM linked to l‐kynurenine metabolism
PBMCs	IM	scRNA‐seq + scVDJ‐seq/10x Genomics single‐cell RNA 5' v2 + V(D)J enrichment	Ref. [74]	Available upon request	3 IM samples; differential cytotoxic and immunoregulatory signatures in CD16/CD56‐stratified NK cell subsets; clonal expansion of EBV gp350‐specific B cells
Liver‐resident B cells	Primary Sclerosing Cholangitis (PSC)	scVDJ‐seq + bulk TCR‐seq + bulk PhIP‐seq/10x Genomics single‐cell RNA 5' v1.1 + V(D)J enrichment	Ref. [69]	Restricted access	3 PSC liver biopsy B cell samples; higher anti‐EBV B cell response specificity in PSC versus primary biliary cholangitis (PBC); bulk TCR repertoire enrichment against EBV antigens (BZLF1, EBNA3A, EBNA1)
PBMCs	Multisystem inflammatory syndrome in children (MIS‐C; SARS‐CoV‐2 sequelae)	scRNA‐seq, scVDJ‐seq/10x Genomics single‐cell RNA 5' v2 + V(D)J enrichment	Ref. [75]	SRA: SRP485868; GEO: GSE254179	11 acute MIS‐C samples; significant upregulation of TGF‐beta signaling across T, B, and monocyte subsets in MIS‐C; TGF‐beta impairs virus‐reactive T cells and cytotoxicity; expansion of (impaired) EBV‐specific TCR clones (TCRVb21.3^+^; similarly expanded by EBNA2 epitope exposure); EBV reactivation (induced by TGF‐beta) is characteristic of MIS‐C
PBMCs	Type 1 Diabetes	scRNA‐seq/10x Genomics single‐cell RNA (capture chemistry not stated)	Ref. [70]	SRA: SRP516833; GEO: GSE271063	14 T1D samples (6 EBV seropositive); improved treatment response (teplizumab, anti‐CD3 antibody) in EBV^‐^ seropositive individuals (lower probability of developing T1D, longer time to disease); EBV presence modifies T cell differentiation trajectories

Spatial‐omics					
Clinical samples: Cancer/lymphoproliferative disease					
Tumor cell of origin	Disease	Assay(s)	Study	Data access	Notes
B cell	DLBCL	Sequencing spatial transcriptomics/10x Visium HD + imaging spatial transcriptomics/Nanostring GeoMx, CosMx + imaging spatial proteomics/CODEX	Ref. [77]		30 DLBCL samples (17 EBV^+^); enrichment of immunosuppressive (M2‐like) macrophages in EBV^+^ DLBCL TMEs; increased T cell exhaustion markers (reduced MHC class II expression, increased PD‐L1 expression in CD4^+^ T cells); macrophage‐CD4‐tumor immunosuppressive axis correlated to EBV LMP1 expression
B cell	DLBCL	Sequencing spatial transcriptomics/10x Visium V2	Ref. [78]	Bio‐Med Big Data Center: OEP00004742	8 DLBCL samples (4 EBV^+^); PD‐1/PD‐L1‐TLR4 immunosuppressive axis in EBV^+^ DLBCL TMEs
B cell	HIV‐associated non‐Hodgkin Lymphoma (HIV‐NHL)	Sequencing spatial transcriptomics/Visium V2	Ref. [79]	GEO: GSE274051	10 HIV‐NHL samples (5 EBV^+^); immunosuppressive SLC40A1^+^ myeloid signature in microenvironments in EBV^+^ tumors from multiple cells of origin; enriched oncogenic axes in EBV^+^ tumors‐TMEs including CD44‐SPP1, GAS6‐AXL; method for spatial gradient analysis relative to annotated tissues/cell types
NK/T cell	NKTCL	Stereo‐seq + scRNA‐seq	Ref. [80]	CNGBdb CNP0005654; BIG GSA: HRA007314	13 NKTCL; myeloid cell enrichment in TME; M2‐like myeloid polarization and enriched immune checkpoint molecule expression; SPP1‐CD44 interaction between TME and malignant cells; elevated LAG3^+^ CD8^+^ T cells in TME; tumor subtype with high MYC activity and fatty acid metabolism signature associated with poor prognosis
Epithelial cell	NPC	Sequencing spatial transcriptomics/Visium V2; stereo‐seq; scVDJ‐seq	Ref. [81]	BIG GSA: HRA006885; GEO: GSE206245	15 NPC samples; tertiary lymphoid structure form within NPC and yield tumor‐infiltrating T and B cells (disease stage‐dependent), including Ab‐secreting plasma cells; tertiary lymphoid structure (TLS) correlation to disease prognosis, with malignant cell inhibition of Ab production and anti‐tumor immunity
Multiple (lymphoid, epithelial cells)	IM	Imaging spatial proteomics/Akoya Phenocycler [CODEX], OPAL multiplexed IF + imaging spatial transcriptomics/Nanostring CosMx	Ref. [82]	Available upon publication	8 IM tonsils (OPAL mIF); 4 IM tonsils (Phenocycler); detection of EBV^+^ tonsillar epithelial cells (differentiated squamous phenotype); spatially resolved EBV latency programs (IIb, III, IIa); prevalence of EBV^+^ B cells outside of germinal centers; evidence for LMP1‐mediated immune microenvironment conditioning; enrichment of exhausted T cell biomarkers (LAG3^+^ TIM3^+^ near LMP1^+^ B cells; TIGIT^+^, ICOS^+^, and TCF1^+^ T cells also in LMP1^+^ regions)

### 2.1 Experimental Models – B Cells

EBV‐infected B cells bear historical significance in the single‐cell transcriptomics field since virus‐positive lymphoblastoid cell lines (LCLs) were used for an early technological demonstration of biological diversity masked in ensemble populations [17]. While groundbreaking, this study only assayed 92 genes, none of which were viral. The first transcriptome‐wide scRNA‐seq study of LCLs likewise focused on cellular expression [18], though subsequent analysis alongside newly‐generated LCL scRNA‐seq data aligned to a multispecies genome identified correlated EBV‐host phenotypic heterogeneity [19]. In addition to rare lytic cells, major axes of heterogeneity in LCLs derive from donor‐dependent clonal heterogeneity propagated through transformation as well as a phenotypic continuum spanning B cell activation and plasmablast differentiation [19], consistent with the germinal center (GC) model of EBV infection [20, 21, 22]. Subsequent analysis showed that an early activated B cell phenotype within LCLs shares molecular signatures with autoimmune‐associated T‐bet^+^CXCR3^+^ B cells that develop outside of GCs [23]. B cell phenotypes in LCLs infected with Type 1 EBV strains (B95‐8, M81, Mutu) are broadly concordant with those identified in Type 2 (BL5) LCLs; however, many cellular genes are differentially regulated by Type 1 vs Type 2 EBV, and phenotype frequencies differ by strain [24]. Notable type‐dependent differences include the increased presence of differentiated plasma cells and late lytic cells with low IRF4 expression in Type 2 LCLs [24]. Subsequent re‐analysis of these datasets confirmed key findings and underscored the role of EBV super enhancer (EBVSE) target genes in maintaining latent infection [25]. A unique co‐infection dataset of LCLs generated from individuals with inherited chromosomally integrated human herpesvirus 6 (HHV‐6A/6B) is also publicly available [26].

Primary B cell infection with Type 1 EBV (B95‐8) has been investigated with temporal single‐cell multiomics and pseudotime analyses [27, 28]. These studies identified genome‐wide regulatory signatures of restricted infection via innate immune activation and a distinct growth arrest trajectory resulting from viral oncoprotein‐induced hyperproliferation. By contrast, successfully infected B cells rapidly acquired gene regulatory environments resembling tonsillar GC and post‐GC B cell stages as well as an activated precursor (AP) niche from which cells may enter or bypass GC‐dependent development. Cross‐referencing these in vitro phenotypes against tonsillar B cell scRNA‐seq and pseudobulk ATAC‐seq data confirmed their physiologic relevance as well as noteworthy differences (e.g., the absence of *BCL6* expression in EBV^+^ GC‐like states in vitro). Consistent with the implication that EBV elicits GC‐dependent and GC‐independent B cell responses, scRNA‐seq revealed distinct responses to infection – innate‐like pro‐inflammatory expression and upregulation of genes linked to neuroinvasion and neurotropism – in *TBX21*(T‐bet)^+^ *CXCR3* ^+^ B cells with high Fc receptor (e.g., *FCRL4*, *FCFL5*) expression and variable *ITGAX* (CD11c) [27]. Notably, phenotypically similar cells including “atypical” B cells (ABCs) [29] activate and develop outside of GCs and contribute to the pathology of several autoimmune diseases associated with EBV, particularly multiple sclerosis (MS) and systemic lupus erythematosus (SLE) [30, 31, 32, 33]. Subsequent scRNA‐seq studies in humanized mouse models elucidated immune control of primary infection in lymphoid tissues [34] and the in vivo relevance of EBV‐induced ABC responses [35]. Remarkably, EBV infection induced oligoclonal ABCs (*TBX21* ^+^ *CXCR3* ^+^), CXCR3‐mediated B cell neuroinvasion, and subsequent activated *CD4* ^+^ T cell recruitment to the central nervous system (CNS) in mice. Apart from T cell responses, self‐ or cross‐reactive ABCs may produce pathogenic antibodies in vivo [35]. Functional single‐cell studies of EBV‐infected B cell migration in transwell assays further demonstrated virus‐induced B cell motility and extravasation (specifically, endothelial diapedesis), which may be important for CNS infiltration [36]. Collectively, single‐cell analyses revealed primary infection‐driven B cell responses that support refined conceptual models for infection and identified an EBV^+^ niche likely critical to virus‐associated autoimmunity.

Recently, single‐cell sequencing was paired with primary infection using recombinant EBV strains to reveal novel roles of EBNA‐LP [37]. This functional application of scRNA‐seq identified increased frequencies of early activated precursor (AP) B cells (CCR6^+^/CD23^lo^; a pre‐GC state) and innate antiviral restriction states in cells infected with EBNA‐LP knockout (LPKO) EBV. Gene regulatory network (GRN) analysis of genes upregulated in LPKO infection predicted a role for EBNA‐LP in suppressing antiviral responses mediated by *SP100* and *SP140L*, which were subsequently validated by orthogonal methods. SP140L‐mediated restriction was also enhanced in cells infected with herpesvirus saimiri (HVS) lacking the ORF3 protein, indicating conserved interference with SP140L‐mediated innate immune restriction across herpesviruses [37]. This study highlights the exciting potential of pairing single‐cell analyses with functional genetic perturbations to discover previously unknown biological functions of viral gene products.

Lytic EBV infection has also been studied with time‐resolved scRNA‐seq of inducible BL cell lines [38]. Resistance to lytic cycle activation correlated with MYC levels (mechanistically established by prior work [39]) and mitotic signatures. Expression and GRN analyses predicted enhanced NF‐κB signaling and IRF3 transcriptional activity in abortive lytic cells, while lytic progression evident from viral transcripts was accompanied by unexpected activation of lineage‐ectopic transcription against the background of global host gene shutoff [40, 41]. RNA expression of two such activated genes (*SOX2*, *ALDH1A1*) in a subset of *BZLF1* ^+^ cells was validated by RNA flow‐FiSH, another single‐cell method [38]. While aberrant host RNA reads in lytic cells can originate from transcriptional readthrough into downstream genes [42], lytic infection by other herpesviruses appears to involve *bona fide* functional host reprogramming [43, 44]. The biological importance of similar reprogramming during EBV reactivation remains to be determined.

### 2.2 *In Vitro* Experimental Models – Epithelial Cells

Single‐cell sequencing studies of EBV infection in epithelial cells have been comparatively limited, possibly due to technically challenging experimental models. A noteworthy study applied scRNA‐seq to analyze a 3D air‐liquid interface (ALI) model of the pseudostratified nasopharyngeal epithelium co‐cultured with lytic B cells [45]. Following B cell removal and single‐cell epithelial preparation, EBV transcripts were detected at low levels in most epithelial cell types. Suprabasal epithelial cells exhibited the clearest lytic signature while basal, ciliated, and mucosecretory cells displayed non‐productive viral programs. Recently, lytic induction treatment in the NPC43 cell line was assayed by time‐resolved scRNA‐seq [46]. A cell population designated as non‐responsive to lytic induction with phorbol ester displayed upregulated *SOX2* and *NTRK2* – genes also observed in NPC clinical samples and associated with cancer stem cells (CSCs). The presented data implied lytic reactivation from differentiated epithelial cell subsets, though read alignment to a human‐only reference (hg38) precludes direct viral phenotyping [46].

### 2.3 Emerging Themes From EBV Model Single‐Cell Sequencing

Bulk cell assays are well‐suited to study robust phenotypes. Single‐cell resolution techniques are essential to deconvolve these phenotypes and to discover subtle yet biologically meaningful variations in cell populations. The intrinsically dynamic nature of EBV infection, even in simple experimental models, is a major source of such variation; resolving it offers new views of foundational conceptual models of infection. In particular, single‐cell sequencing can uncover niche‐dependent viral and host programs likely relevant to EBV‐associated pathology. For example, the intersection of EBV and T‐bet^+^ B cells sheds new light on the potential for GC‐independent viral access to the latent memory reservoir. This supports an update to the GC model [22, 47] and may be a key to investigating viral mechanisms in autoimmunity. Diverse host‐virus phenotypic states observed in vitro may recapitulate tumor cell heterogeneity in certain EBV‐positive malignancies and thus provide models for therapeutic response and resistance. Likewise, single‐cell observations of stem‐like plasticity in B and epithelial cell models underscore EBV‐mediated transcriptional reprogramming as a potential viral oncogenic mechanism. In principle, any aspect of infection that can be faithfully modeled (and perturbed) in vitro or in vivo can be assayed with single‐cell sequencing. The question of whether to do so ultimately depends on experimental scope (discovery vs. targeted hypothesis testing), *a priori* knowledge of phenotypes of interest, and practical considerations (e.g., cost, availability of existing data resources).

### 2.4 Clinical Samples – Lymphoid Malignancies

Several single‐cell investigations of B cell malignancies have been reported to date. Enrichment of Th17‐polarized *CD4* ^+^ T cells in was identified in EBV^+^ classic Hodgkin Lymphoma (cHL) in addition to *LAG3* ^+^ T cells with downregulated MHC‐II expression independent of EBV status [48]. One EBV^+^ sample in a scRNA‐seq study of diffuse large B cell lymphoma (DLBCL) subtypes harbored a distinct non‐malignant memory B cell population characterized by elevated *CD44* expression [49], though specific insights on infected tumor cells were limited. A recent scRNA‐seq examination of BL in people living with HIV (PLWH) described intratumoral viral heterogeneity beyond the conventional designation of BL as expressing the latency I program [50]. Peripheral blood mononuclear cell (PBMC) scRNA‐seq from pediatric liver recipients with post‐transplant lymphoproliferative disorder (PTLD) identified dysfunctional memory B cells (uninfected bystanders), increased frequencies of EBV^+^ B cells, immunosuppressive T_regs_ (attributed to immunosuppression treatment), and exhausted CD8^+^ T cells (attributed to chronic EBV infection) relative to uninfected and healthy EBV^+^ donors [51]. EBV‐associated NK/T cell lymphoma (NKTCL) has also been studied with scRNA‐seq, wherein elevated expression of the chemokines *CCL3*, *CCL4*, and *CCL5* in malignant NK cells suggested immune cell chemoattraction to tumors [52]. However, tumor microenvironment (TME) immunosuppression signatures, particularly in T cells, were associated with malignant LMP1^+^ NK subsets. A brief report detailing viral capture enrichment for NKTCL likewise described EBV infection in multiple T cell subsets from nasopharyngeal biopsies [53]. Though limited, these studies indicate EBV‐mediated intratumoral diversity and disease‐specific immunomodulation in lymphoid malignancies.

### 2.5 Clinical Samples – Epithelial Malignancies

Nasopharyngeal carcinoma (NPC) tumor and tumor microenvironment (TME) heterogeneity have been investigated extensively with scRNA‐seq. Interestingly, known sub‐chromosomal copy number variation (CNV; e.g., chr11q and chr12p duplication and chr3p deletion) in malignant NPC cells can be recovered from scRNA‐seq read density binned by genomic region [54, 55, 56, 57, 58]. Studies that employed multispecies reference alignment to detect EBV reads identified co‐expression of latent and lytic program genes within individual tumor cells, particularly read enrichment from the viral genome (fused) termini (e.g., the latent membrane proteins and BART miRNAs). NPC tumor cells also appear to be variably susceptible to infection based on viral receptor expression (*EPHA2*) and cell differentiation status [54, 55, 58]. These insights imply the importance of non‐canonical infection or otherwise abortive lytic reactivation in certain NPC tumor niches. Notably, an abortive lytic program has been linked with recruitment of tumor‐promoting immune cell populations i*n vitro* [59]. Clonal expansion of tumor‐infiltrating lymphocytes (primarily T cells) and immune response exhaustion signatures are generally conserved in TME from NPC samples. Exhausted subsets frequently enriched in NPC TMEs include *CD4* ^+^ T cells (*PDCD1* ^+^ *TIGIT* ^+^) and *CD8* ^+^ T cells (*LAG3* ^+^ *HAVCR2(TIM3)* ^+^), consistent with dysfunctional immune control of tumor cells. Exhaustion associated with chronic activation was most clearly illustrated by upregulated cytotoxic effector genes (e.g., granzyme isoforms) in expanded *CD8* ^+^ *LAG3* ^+^ *HAVRC2* ^+^ T cells. Immunosuppressive functions of TME‐resident regulatory T cells (T_reg_, *FOXP3* ^+^) were identified via cell‐cell communication prediction. Analysis of tumor‐infiltrating B cells in one study [54] found the intriguing presence of *TBX21* ^+^ *FCRL4* ^+^ B cells exhibiting signatures of STAT‐NF‐κB signaling and broad IRF upregulation (*e.g., IRF1, IRF7, IRF8, IRF9*) within the NPC TME. Similarly, activated B cell infiltrates with strong interferon‐induced profiles and plasma cells are enriched in NPC TME versus non‐malignant biopsies and correlate with improved survival [57, 58]. Tumor infiltration by these B cells as well as pro‐inflammatory dendritic cell (DC) and macrophage subsets appear to be enhanced in tumors with detectable EBV. Analysis of scVDJ‐seq data showed that T cells exhibiting activated‐to‐exhausted transition and interferon‐responsive B cells in the NPC TME are clonally expanded, including clones that recognize EBV antigens [57, 58]. Thus, EBV infection shapes complex pro‐ and anti‐tumor immune responses across infiltrating lymphoid and myeloid niches in NPC beyond directly mediating infected tumor cell malignancy.

Single‐cell sequencing studies of other EBV‐associated epithelial cancers remain extremely limited. For example, a scRNA‐seq survey of gastric adenocarcinoma (GA) included one EBV^+^ case without direct viral transcript detection. A cluster of cells expressing *LY6K*, elevated MHC class II genes, and antigen processing gene ontology enrichment was unique to the EBV^+^ case [60], though the definitive identity and viral status of such cells is unclear. A more recent study applied scRNA‐seq and scVDJ‐seq to investigate treatment response of GA stratified by EBV status [61]. EBV^+^ GA displayed elevated lymphocyte infiltration (B and T cell subsets) relative to virus‐negative tumors. Similar to NPC, tumor‐infiltrating T cells in EBV^+^ GA included enriched *CD8* ^+^ *LAG3* ^+^ cells indicative of exhausted/non‐responsive states associated with chronic infection. Co‐expression of *ISG15* in this T cell niche further indicated antiviral interferon response activation. Immunotherapeutic disruption of LAG3‐mediated immune checkpoint led to favorable clinical responses in EBV^+^ GA including reduced tumor burden [61], highlighting the clinical value of targeting EBV‐mediated tumor‐immune axes in epithelial carcinomas.

### 2.6 Clinical Samples – Autoimmune and Inflammatory Diseases

Increasing evidence for roles of EBV in multiple sclerosis (MS) and other autoimmune diseases has broadened clinically relevant applications of single‐cell sequencing for viral studies. Early scRNA‐seq analysis of PBMCs and cerebrospinal fluid (CSF) from people with MS (PWMS) identified expansion of neuroinvasive pro‐inflammatory *TBX21* ^+^ *CXCR3* ^+^ B cells, though no EBV transcripts were detected [31]. Shortly after the publication of Bjornevik and colleagues' landmark study supporting EBV etiology in MS [32], Lanz and colleagues reported BCR cross‐reactivity between EBNA1 and the neuronal surface receptor GlialCAM in a subset of PWMS identified from scVDJ‐seq analyses [33]. Subsequent scRNA‐seq and CITE‐seq studies of PBMCs [62] and cervical lymph node (cLN) aspirates from PWMS [63] have confirmed the pathogenic relevance of ABCs and DN2 B cells linked to EBV infection. Early MS disease activity following baseline diagnosis was significantly associated with a proinflammatory and neuroinvasive transcriptional signature in ABCs [62]; these and several additional B cell subsets identified within cLN of PWMS exhibited host biomarkers of lytic reactivation, which may subsequently drive expansion of EBV‐recognizing CD8^+^ T cells [63]. In these and prior studies, it bears emphasis that the lack of direct EBV detection with scRNA‐seq is commonplace. This likely reflects significant technical limitations (e.g., sequencing depth) as well as the biological rarity of EBV^+^ cells within the B cell reservoir and low viral transcript abundance.

Additional autoimmune disorders are clinically associated with EBV infection [64], though few scRNA‐seq studies of these diseases have directly or indirectly investigated viral roles. PBMC analyses identified enrichment of *CD56* ^‐^ *CD16* ^+^ *FCER1G* ^‐^ NK cells exhibiting an antiviral response in primary Sjögren's Syndrome (pSS) versus healthy control samples [65]. A study of systemic lupus erythematosus (SLE) PBMCs accounting for active EBV infection identified possible expansion of *CD8* ^+^ T cells and monocyte depletion in EBV^+^ cases, though limited sample sizes (*n* = 2 EBV^+^ *vs. n* = 4 EBV^‐^ SLE samples) precluded conclusions of statistical significance. Distinct transcriptional profiles in *CD4* ^+^ T_regs_ and NK cells were also reported in EBV^+^ SLE [66]. Sequencing reads in each of these studies were aligned against human‐only reference genomes. By contrast, a recent study demonstrated EBV‐reprogrammed antigen presentation in SLE autoreactive B cells enabled by multispecies mapping in conjunction with single‐cell enriched viral read capture [67]. Analysis of synovial fluid samples from people with rheumatoid arthritis (RA) using scVDJ‐seq showed no statistically significant relation between lymphocyte clonality and EBV, though seven of twelve patient samples contained multiple T cell clones with cognate EBV antigens. EBV‐specific TCR sequences reactive with latent (EBNA1, EBNA3A, EBNA3B) and lytic (BZLF1, BMLF1) proteins were observed; BZLF1 and BMLF1 epitopes were most commonly recognized across individuals [68]. Recent scVDJ‐seq analysis of PBMCs from people with primary sclerosing cholangitis (PSC), a chronic autoinflammatory liver disease, established a significant clinical association with EBV but not with other common infectious agents [69]. Enriched T cell clones primarily exhibited reactivity against BZLF1, EBNA3A, and EBNA1 versus controls. Enriched EBV‐targeted antibody responses, particularly against BMRF1 epitopes, indicated immune responses against lytic EBV infection are elevated in people with PSC [69]. In contrast to viral pathogenicity, scRNA‐seq analysis of type 1 diabetes treatment response anti‐CD3 monoclonal antibody identified unexpected immunotherapeutic response enhancement by latent EBV infection [70].

EBV‐associated conditions characterized by chronic and/or severe inflammation have likewise been dissected with single‐cell sequencing. PBMC landscapes from Infectious Mononucleosis (IM), Chronic Active EBV (CAEBV), and Hemophagocytic Lymphohistiocytosis (HLH) underscore T and NK cell inflammatory responses in poorly‐controlled EBV infection [71, 72, 73, 74]. IM PBMCs included virus‐positive clonally expanded B cells (some co‐expressing latent and lytic antigens), proinflammatory T and NK subsets, and an immunoregulatory NK niche [74]. CAEBV PBMCs exhibited strikingly broad infection across myeloid and lymphoid lineage cells as well as hematopoietic stem cells (HSCs), possibly indicating disease origin from an infected multipotent hematopoietic progenitor [72]. Hematopoietic stem cell transplantation (HSCT) in one enrolled individual with CAEBV led to inflammatory symptom abatement and undetectable EBV infection [72]. In HLH, hyperactive type I and type II interferon responses were observed in several T, NK, and myeloid cell populations [71, 73]. *CD8* ^+^ T cell exhaustion (*LAG3* ^+^) was observed in HLH PBMCs, as in other EBV‐associated cancers and autoimmune diseases [71]. Data from one study indicated that monocyte differentiation into pro‐inflammatory states observed in HLH is linked to IDO1‐mediated metabolism of l‐kynurenine [73]. This metabolic signature was depleted in individuals exhibiting complete response to standard HLH treatment; however, the potential viral role in this monocyte metabolic activity is presently unknown. Finally, T cell scRNA‐seq analysis of multisystem inflammatory syndrome in children (MIS‐C) following SARS‐CoV‐2 infection revealed a critical role for EBV reactivation and virus‐specific immune responses [75]. Transforming growth factor beta (TGFβ) appears to play a dual pathogenic role in this disease by inducing EBV reactivation from latency [76] and impairing virus‐directed T cell cytotoxicity. Interestingly, EBV‐reactive CD4^+^ and CD8^+^ T cells from MIS‐C patients consistently recognized EBNA2 epitopes in vitro [75].

### 2.7 EBV Clinical Sample Spatial‐Omics

The field of EBV spatial transcriptomic and proteomics is still in its infancy but can be expected to grow exponentially in the near future. To date, high‐dimensional spatial profiling techniques have been used to investigate cancers including EBV‐stratified DLBCL and other non‐Hodgkin lymphomas [77, 78, 79], NKTCL [80], and NPC [81]. High‐resolution spatial multiomics of cellular and viral biomarkers recently revealed EBV‐induced B cell heterogeneity and immune interactions in tonsils from individuals with acute IM [82]. Along with immunosuppressive polarization of macrophages, T cell dysfunction and exclusion from EBV^+^ B cell interaction have been observed frequently in these studies. Within IM tonsils, immunosuppressive microenvironmental features spatially correlate with LMP1‐expressing B cells outside of GCs [82]. Current and future spatial‐omics data will thus likely be valuable guides for clinical management of EBV‐associated diseases.

### 2.8 Emerging Themes From EBV Clinical Sample Single‐Cell and Spatial Sequencing

High‐resolution studies of EBV‐associated disease indicate the clinical relevance of intra‐disease infection heterogeneity and virus‐conditioned immune responses. For example, tumors conventionally classified as displaying a uniform infection program often contain infected cell populations across a range of latent and lytic stages. Viral transcriptional diversity is likely key to pathogenesis because EBV gene programs distinctly affect biological functions of infected cells. For example, the GC‐independent EBV^+^ T‐bet^+^ B cell niche found via single‐cell approaches reflects lymphocytes with low antigen affinity and high innate‐like inflammatory potential and thus represents a clinically important target in autoimmune diseases. Across cancers and autoimmune diseases, EBV‐infected cell heterogeneity appears instrumental in shaping immune recognition, response, and evasion across cell‐cell interaction axes. In virus‐positive tumors, specific mechanisms of EBV‐mediated immune suppression and evasion may correlate with viral expression programs and accordingly vary across tumor microenvironment neighborhoods. It is noteworthy that cytotoxic T cell exhaustion, immunoregulatory T cell enrichment, and immunosuppressive myeloid cell skewing are generally conserved in distinct EBV‐associated cancers of distinct cellular origins and in certain inflammatory disorders. Several of these immune features appear to reflect defective control of EBV reactivation and chronic responses to infection. Collectively, single‐cell and spatial insights into the precise cell‐cell molecular interactions underlying EBV‐associated diseases may prove extremely valuable for informing effective and tailored chemo‐ and immunotherapies. Discoveries of previously unknown EBV roles in PSC and MIS‐C benefited from single‐cell approaches, underscoring the possibility that additional unexpected EBV associations to diseases might be similarly revealed in the future. However, technical improvements in viral read detection sensitivity and routine implementation of EBV sequence alignment continue to be major priorities in future studies and analyses of existing data.

### 2.9 Future Directions in Single‐Cell EBV Virology

#### 2.9.1 Technological Challenges and Opportunities

**Figure 4:**
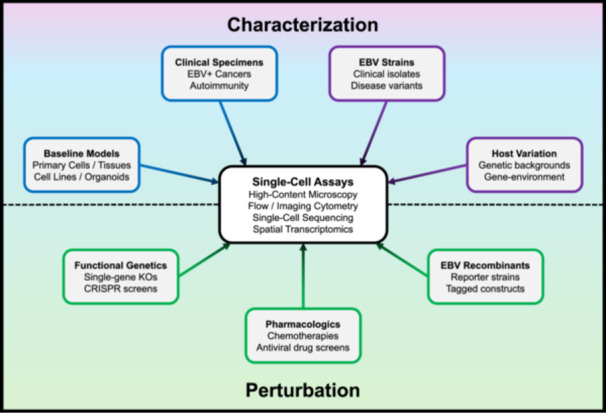
Experimental designs for EBV infection models and tissues upstream of single‐cell assays.

Single‐cell experimentation contributed to the foundations of the EBV research field. Contemporary single‐cell approaches, which vastly expand the measurable dimensions and scale of biological information obtained from infection models and clinical samples, have been and will likely continue to be indispensable in understanding and addressing EBV‐associated diseases. Single‐cell and spatial technologies provide increasingly comprehensive assays spanning characterization and comparison to perturbation and mechanistic studies of infection (Figure 4). A substantial body of data characterizing EBV models and clinical specimens already exists, and more is certain in the near future. The extent of useful biological information within one single‐cell or spatial sequencing dataset is virtually impossible to explore within a single study; in other words, much remains to be discovered through careful mining of existing resources in the field. Re‐mapping existing sequence data to detect viral reads may be effective in certain cases, whereas *de novo* experiments implementing viral read capture strategies (e.g., spike‐in sequences for hybridization probe libraries or beads with custom capture sequences in drop‐seq) [53, 67] will be required in other instances. Even so, read dropout inherent to single‐cell assays can obscure the identification of truly virus‐negative cells versus technical false negatives. Dropout may likewise impair accurate annotation of infection stages in cells with detected viral reads, particularly in latently EBV‐infected cells due to low overall viral expression levels and dependence on combinatorial gene profiles to delineate different programs (latency I, IIa, IIb, and III). Reliably matching clinical disease features to precise EBV infection programs and gene functions depends on correcting this technical limitation. Thus, direct and sensitive (deeply sequenced) single‐cell viral read detection represents a critical long‐term goal for the field.

While original sequences from many EBV single‐cell and spatial datasets are publicly available (Table 1), improving the accessible use of these resources should be a major current and future priority. This can be achieved via two parallel efforts: 1) creation and curation of an interactive exploratory database for EBV infection model and clinical sample datasets that lowers the barrier to entry for researchers without extensive programming or bioinformatics experience; and 2) ongoing training and collaborative efforts to increase familiarity and use of advanced single‐cell and spatial analyses throughout the field.

Apart from leveraging current data, expanding single‐cell applications beyond characterization to studies with functional genetic control and pharmacologic screening is a critical avenue for the field. High‐throughput compound screening at single‐cell resolution will likely provide an engine for antiviral compound discovery and testing. Combining single‐cell methods with tractable viral and host genetics including gene reporters, knockouts, silencing, and activation offers unprecedented opportunities to reveal fundamental mechanisms and outcomes of infection, the genetic basis of host‐pathogen dynamics, and their disease relevance. Again, the success of such approaches will require complementary expertise in experimental models of infection and computational informatics – particularly through collaborative efforts.

Perhaps the single greatest barrier to the uptake of single‐cell and spatial experiments is cost. The price of commercially available assays rapidly becomes prohibitive, which poses a particular challenge to making statistically powered comparisons. Development of more cost‐effective technologies will help mitigate this issue and is especially vital for researchers in low‐resource settings. Paired use of small‐scale single‐cell pilot studies with higher throughput orthogonal methods may provide a practical alternative for some studies. Sequencing‐informed design of flow cytometry and/or quantitative microscopy panels are especially appealing for this purpose. In principle, thoughtful design and execution of single‐cell sequencing, cytometry, and imaging assays for a given infection model could facilitate cross‐platform data integration. Such unification of many single‐cell modalities would provide remarkable perspectives of gene regulation within individual EBV‐infected cells, the immune responses they elicit, and their significance to viral diseases.

#### 2.9.2 Open Questions in Molecular Mechanisms of EBV Pathogenesis

**Figure 5:**
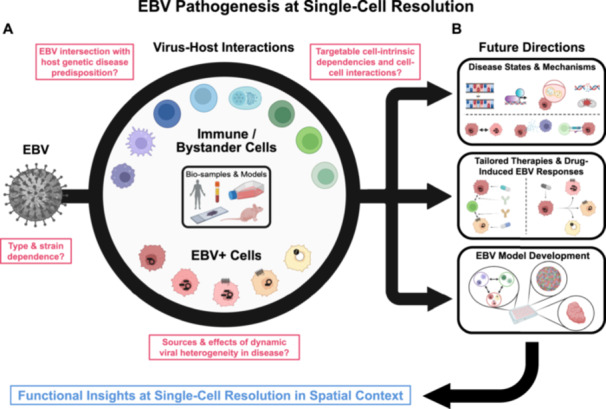
Open questions and future directions for single‐cell studies of EBV pathogenesis. (A) Genome‐wide phenotypes and interactions of EBV‐infected cells (bottom half of circle: latent and lytic‐infected cells in red hues with EBNA and LMP profiles depicted) and bystander cells (top half of circle: myeloid cell types in purple hues, B cell types in blue hues, NK/T cell types in green hues) within experimental models and clinical disease specimens (middle of circle: patient blood and tissue biopsies, cultured cells, murine models) can be revealed by single‐cell and spatial approaches. These assays and analytic tools are well‐suited to address open questions (red boxes) in the biology of EBV‐associated cancers and autoimmune diseases. (B) Future single‐cell and spatial applications in EBV research will help define disease‐specific cell states and mechanisms (top row, cell‐intrinsic: disease‐associated genetic variants, gene regulation, metabolic activities and cell stressors; cell‐extrinsic: dynamic infection states, paracrine EBV‐bystander signaling, direct EBV‐bystander cell interactions); identify effective therapies tailored to specific EBV‐associated diseases (middle row, left: pharmacologic and biologic targeting of distinct infected cell states and cell‐bystander interactions) as well as viral responses to treatment (middle row, right: drug‐induced changes in viral expression program and host cell biological state); and provide high‐resolution readouts to support development of improved experimental models of EBV‐associated diseases (bottom row: co‐culture systems, tumor organoids, and *ex vivo* whole tissues). *(Figure created with BioRender under license)*.

Distinct EBV‐positive malignancies characteristically exhibit predominant infection programs [83], though single‐cell and spatial studies have rendered more nuanced landscapes of intratumoral viral expression heterogeneity. While diverse EBV infection programs [84] have been dissected in vitro and correspond to biologically distinct host cell states [21, 27, 85], the physiologic origins and clinical consequences of intratumoral EBV heterogeneity are not definitively clear. Taking an example from Burkitt Lymphoma, where the majority of EBV^+^ malignant cells characteristically exhibit latency I (EBNA1^+^EBNA2^‐^LMP1^‐^), do rare EBNA2^+^ malignant cells [79] reflect EBV genome responses to the programming of a host cell (perhaps an infrequent but developmentally distinct B cell state) or merely stochasticity in viral promoter usage? Do multiple EBV infection programs within a tumor represent static states or dynamic viral transitions *in situ*? Understanding intratumoral viral heterogeneity is a high priority since EBV expression patterns can influence infected cell interactions with bystanders, and immune cell composition and functions likely influence viral expression programs in turn [86]. Analogous to emerging themes in EBV‐associated autoimmune diseases [33, 63, 67], the effects of EBV strain, host genetic background, and gene‐environment interactions are especially important yet understudied factors that likely shape not only the risk for EBV‐associated cancers but also their clinical presentations. Single‐cell and spatial methods provide valuable tools to address these unanswered questions in principle by resolving niche‐specific hierarchical regulation in host‐virus interactions (Figure 5A).

Answering these and other questions is critically important to understand EBV‐specific disease mechanisms, tailor clinical therapies, and inform new models for dissecting disease‐relevant aspects of infection (Figure 5B). For example, in vitro lymphoma models with different cell backgrounds and viral expression programs exhibit shared and distinct growth dependencies [87]. The mechanistic actions of viral genes in concert may present distinctive cell‐intrinsic and/or immune response vulnerabilities that are not exploited by current clinical therapeutic regimens for EBV‐positive malignancies. Conversely, treatments may select for changes in viral programs and replication that complicate clinical responses. Even the most detailed single‐cell and spatial‐omics studies to date in the EBV field capture snapshot clinical specimens that are intractable for interrogating viral dynamics and treatment responses. This reflects the limited availability of longitudinal EBV^+^ cancer samples spanning initial diagnosis through therapeutic course and imposes a practical constraint to addressing key questions in EBV pathogenesis. Thus, developing new high‐fidelity models coupled with high‐dimensional assays and bioinformatic inference techniques will be essential. Ambitious pairing of single‐cell and spatial‐omics analyses with prospective clinical studies at scale is logistically challenging; however, such designs may be vital for resolving the mechanistic logic of EBV pathogenesis in cancers and autoimmunity. When the time presents itself, single‐cell and spatial investigations of EBV will be indispensable to break the code, treat the diseases [1].

## Author Contributions

**Elliott D. SoRelle:** conception, creation, and edits of all text, figures, and tables.

## Conflicts of Interest

The author declares no conflicts of interest.

## Supporting information

2025_JMV_single_cell_EBV_SUPP_rev_1.

## Data Availability

Data sharing not applicable to this article as no datasets were generated or analysed during the current study.
