# Supplementary material for: Epstein‐Barr Virus Infection at Single‐Cell Resolution
Source: J Med Virol. 2026 Feb 5;98(2):e70825. doi: 10.1002/jmv.70825 (PMC12875166; doi:10.1002/jmv.70825)
Supplement: Supplementary file 1 — 2025_JMV_single_cell_EBV_SUPP_rev_1. [file JMV-98-e70825-s001.pdf]

Supplementary Text

Epstein-Barr virus infection at single-cell resolution

Elliott D. SoRelle[1,2]

[1] Department of Microbiology & Immunology, University of Michigan, Ann Arbor, MI 48109

[2] Department of Biological Chemistry, University of Michigan, Ann Arbor, MI 48109

Corresponding Author

Elliott D. SoRelle, PhD

sorelle@med.umich.edu

Keywords

Epstein-Barr virus, EBV, herpesviruses, virology, single-cell, scRNA-seq, scATAC-seq, spatial -omics, flow cytometry, high-content screening, microscopy, cancer, oncogenesis, B cell lymphoma, nasopharyngeal carcinoma, NK/T cell lymphoma, infectious mononucleosis, autoimmunity, multiple sclerosis

GLOSSARY

ABC: atypical B cell
Akata: Burkitt Lymphoma-derived cell line
AITL: Angioimmunoblastic T cell lymphoma
ALI: air-liquid interface
AP: activated precursor (B cell state)
ATAC-seq: assay for transposase-accessible chromatin sequencing
BCR: B cell receptor
BL: Burkitt Lymphoma
CAEBV: chronic active EBV
cDNA: complementary DNA
ChIA-PET: chromatin interaction analysis with paired-end tagging
cHL: classic Hodgkin Lymphoma
CITE-seq: cellular indexing of transcriptomes and epitopes sequencing
cLN: cervical lymph node
CNV: copy number variation
CODEX: co-detection by indexing
CosMx: NanoString platform for spatial imaging transcriptomics
CSC: cancer stem cell
CSF: cerebrospinal fluid
DE: differential expression / differentially-expressed (as in genes)
DLBCL: diffuse large B cell lymphoma
DN2: double-negative type 2 cells (IgD-CD27- B cell population)
drop-seq: droplet-based single-cell library preparation technique
EBER: EBV-encoded small RNAs
EBER-iSH: EBER *in situ* hybridization
EBNA: EBV nuclear antigen (viral latency genes)
EBV: Epstein-Barr virus (HHV-4)
EBVSE: EBV super enhancer (gene regulatory complex)
ELISA: enzyme-linked immunosorbent assay
FACS: fluorescence-activated cell sorting
Flow-FiSH: flow cytometry fluorescence *in situ* hybridization
GA: gastric adenocarcinoma
GC: germinal center
GRN: gene regulatory network
HHV: human herpesvirus

Hi-C: chromatin conformation capture technique

HIV: human immunodeficiency virus

HIV-NHL: HIV-associated non-Hodgkin lymphoma

HL: Hodgkin Lymphoma

HLA: human leukocyte antigen

HLH: hemophagocytic lymphohistiocytosis

HRS: Hodgkin Reed-Sternberg (HL malignant cell)

HSC: hematopoietic stem cells

HSCT: hematopoietic stem cell transplantation

IF: immunofluorescence

IM: infectious mononucleosis

IRF: interferon regulatory factor

ISH: *in situ* hybridization

K-NN: k-nearest neighbors

LCL: lymphoblastoid cell line

LMP: latent membrane protein (viral latency genes)

mRNA: messenger RNA

MHC: major histocompatibility complex

MIS-C: multisystem inflammatory syndrome in children (SARS-CoV-2 sequelae)

MS: multiple sclerosis

NALT: nasopharynx-associated lymphoid tissue (murine equivalent to tonsillar tissue)

NGS: next-generation sequencing

NHL: non-Hodgkin lymphoma

NK cell: natural killer cell

NKTCL: NK/T cell lymphoma

NPC: nasopharyngeal carcinoma

NPC43: nasopharyngeal carcinoma cell line

ORF: open reading frame

PBMC: peripheral blood mononuclear cells

PC: principal component

PCA: principal component analysis

PCR: polymerase chain reaction

PLWH: people living with HIV

PSC: primary sclerosing cholangitis

pSS: primary Sjögren’s syndrome

PTLD: post-transplant lymphoproliferative disorder

PWMS: people with multiple sclerosis

QC: quality control

RA: rheumatoid arthritis

RNA-seq: RNA sequencing

scATAC-seq: single-cell assay for transposase-accessible chromatin sequencing

scChIP-seq: single-cell chromatin immunoprecipitation sequencing

scMNase-seq: single-cell micrococcal nuclease sequencing

scRNA-seq: single-cell RNA sequencing

scVDJ-seq: single-cell variable, diversity, joining segment sequencing (general technique for scBCR/TCR-seq)

SLE: systemic lupus erythematosus

split-seq: plate-based single-cell barcoding library preparation strategy

TCR: T cell receptor

TGF-β: transforming growth factor beta

TME: tumor microenvironment

TR: tandem repeat

t-SNE: t-distributed stochastic neighbor embedding

UMAP: uniform manifold approximation projection

UMI: unique molecular identifier

Visium HD/V2: 10x Genomics spatial transcriptomics platform

VRC: viral replication compartment

I. Single-Cell EBV Virology: A Historical Perspective

A. Early Single-Cell Insights (ca. 1970-2000)

Foundations of EBV Infection in Disease

Limiting dilution cloning: Poisson-limited dilution cloning is arguably the prime example of a powerful early experimental method to study the behaviors of individual infected cells. Such cloning of EBV-positive lymphoid cell lines confirmed viral genome presence in EBV+ lines despite the rarity of cells with detectable lytic antigens[1]. Single-cell cloning further revealed the inefficiency of primary EBV infection (~3% B cell transformation rate) despite the transforming capability of even a single virion[2]; enabled precise quantitation of EBV genomes and their amplification after cellular transformation[3]; and was used to quantify latent viral genome replication and partitioning to daughter cells[4]. Notably, the spontaneous loss of virus in sub-cloned EBV+ Akata BL cells enabled isogenic demonstration of EBV-mediated malignancy independent of virus-induced cell proliferation[5]. Reinfection of EBV- Akata cells later provided a useful system to produce and study recombinant viral strains[6].

EBV genome structures and amplification: Viral genome detection methods likewise played a key role in determining the single-cell origins of EBV-positive cancers. The structure of the circularized viral genome itself[7] provides a reliable indicator of infected cell clonality. Specifically, clones can be identified through conserved numbers of direct tandem repeat (TR) sequences in episomes derived from linear EBV genomes, which exhibit variable terminal TR copies[8]. Viral TR analyses of BL-derived Raji cells and nasopharyngeal carcinoma (NPC) biopsy tissue supported monoclonal malignancy development after infection[8], consistent with viral etiology. Subsequent studies in carcinoma lines found that infected epithelial clones harboring EBV episomes with shorter fused terminal TR regions have a proliferative advantage *in vitro*[9], suggesting the tumors may have begun as virally polyclonal entities that experienced rapid clonal selection. Mechanistically, the inverse relationship between terminal TR length and proliferative advantage may be explained by more efficient expression of the viral oncogene LMP-2A, whose open reading frame is spliced across the viral genome ends[9,10]. Interestingly, TR analysis of EBV-associated post-transplant lymphoproliferative disease (PTLD) identified rare polyclonal (polymorphic) tumors distinct from more common monomorphic lesions[11]. Single-cell PCR amplification of another tandem repeat region (the internal BamHI locus) was used to demonstrate EBV presence in lymph nodes from some individuals with angioimmunoblastic lymphadenopathy (AITL)[12] and within single Hodgkin Reed-Sternberg (HRS) cells of Hodgkin Lymphoma[13,14].

Brightfield and fluorescence microscopy: Microscopy – the original single-cell method[15,16] – quickly proved instrumental for evaluating EBV infection in experimental and clinical samples. For example, microscopy was foundational to the characterization of the B95-8 viral strain and its marmoset-derived producer cell line[17]. Consistent with human EBV+ lymphoid lines, immunofluorescence (IF) detection of viral antigens revealed

relatively few lytic (producer) cells, which intriguingly exhibited large multinucleated phenotypes[17]. Early quantitative microscopic analysis of B95-8 and P3HR-1 EBV demonstrated that viral transforming capacity and lytic infection are independent phenomena[18]. Immunohistochemistry (IHC) and IF microscopy also quickly became indispensable tools to dissect EBV presence and heterogeneity in clinical samples including NPC[19]; AITL[20]; HIV-associated non-Hodgkin lymphomas (HIV-NHL)[21,22]; and infectious mononucleosis (IM)[23]. IHC and IF analyses subsequently confirmed intratumoral viral heterogeneity within *in vitro* BL models[24,25] and a murine PTLD model[26]. Sensitive brightfield detection of EBV via EBER *in situ* hybridization (ISH)[27] is now a routine aspect of cancer diagnosis with suspected EBV involvement, while IHC against viral antigens (e.g., EBNA1, EBNA2, LMP-1, BZLF1, and EA-D) is used for detailed phenotyping.

<u>Other early single-cell studies</u>: Though a full account is not feasible herein, several early single-cell studies merit discussion for their innovative designs. Yarchoan and colleagues coupled limiting dilution cell isolation with enzyme-linked immunosorbent assay (ELISA) to quantify the frequency and isotype-restricted nature of antibody-producing cells after primary B cell infection[28]. Statistical analyses of immunoglobulin (Ig) production with limiting dilutions of virus also confirmed the "one-hit" (single-virion) nature of B cell activation by EBV[28]. In another key study, Thorley-Lawson and Mann used low-parameter flow cytometric analyses of primary B cell infection to define kinetically distinct cell response states[29]. Specifically, small B cells expressing CD23 (BLAST-2, *FCER2*) and upregulated RNA synthesis were found to precede CD23-negative proliferating lymphoblasts. By sorting these cells and detecting viral genomes in each population, this study provided a foundation for the germinal center (GC) model of EBV infection[30], which continues to be refined[31].

<u>B. Advances in Single-Cell EBV Methods (ca. 2000-2020)</u>

Infection Heterogeneity and Dynamics

<u>Advanced single-cell cloning and genome quantification</u>: Single-cell cloning continued to provide powerful means for discerning malignant cells of origin and elaborating the working model of EBV primary infection by host cell niche. Coupling clonal lymphoblastoid cell line (LCL) outgrowth with flow cytometry and IgH_V locus amplification revealed that EBV could rescue BCR-deficient GC B cells otherwise destined for apoptosis, likely through pro-survival latent membrane proteins (LMP-1 and LMP-2A) signaling[32]. Separate clonal analysis of EBV-positive B cell IgH_V sequences and antibody profiling from people with acute IM demonstrated that the virus can persist in self-reactive memory B cells[33]. Interestingly, autoreactive EBV-positive clones did not appear to correlate with pathogenic antibody production during acute IM[33]. In retrospect, the observed viral persistence in clones recognizing self- and non-self-antigens likely reflected the ability of EBV to establish latency via GC-dependent and GC-independent B cell development. Isolating individual infected cells for downstream assays remains a workhorse approach in the field. Notably, clonal isolation is not strictly required to obtain single-cell infection insights. In an exemplary study, Hadinoto and colleagues quantified viral genomes

in saliva and used mathematical modeling to infer shedding dynamics in the oral cavity[34]. In addition to revealing multi-log order variation within a given person over time, quantitative results indicated tonsillar B cells are not the primary source of new virions. As few as ~3 infected epithelial cells at any time may be responsible for EBV particle amplification based on computational modeling[34], though experimental validation remains challenging.

Multiparameter flow cytometry to identify infection niches and dynamics: Fluorescence-activated cell sorting (FACS) achieves single-cell resolution of multiple biomarkers in addition to downstream population recovery for pseudobulk assays. Miyashita and colleagues leveraged these capabilities to identify EBV within resting CD19+/CD20+ peripheral blood mononuclear cells (PBMCs) from healthy donors[35]. The Thorley-Lawson group later applied B cell surface marker sorting and downstream genome detection to definitively establish memory B cells as the classic *in vivo* reservoir[36] and further develop the empirical basis for the GC model by relating latent infection programs to host cell phenotypes[30,37,38]. Similar approaches established the link between B-to-plasma cell differentiation and lytic reactivation[39]. Flow cell cycle analysis revealed cell arrest upon treatment with lytic-inducing agents[40]. Similar methods were used to show that certain p53-deficient EBV-positive lines can bypass drug-induced mitotic checkpoints[41-44]. Combined surface protein, cell cycle, and proliferation tracking stains made flow cytometry an excellent technique for single-cell quantitative studies in primary EBV infection models. Such combinations underscored the existence of diverse post-infection B cell fates by defining transformation barriers including viral genome delivery to the nucleus[45], proliferation-induced DNA damage responses and growth arrest[46], and metabolic stress[47]. As in studies of primary infection, re-culturing sorted cell populations can uncover surprising heterogeneity and infection dynamics in BL-derived cell lines exhibiting high rates of spontaneous lytic infection[48]. Exciting advances coupling flow cytometry with fluorescence *in situ* hybridization (Flow-FiSH) enabled niche-specific sensitive genome detection in clinical samples from chronic active EBV (CAEBV) and virus-associated malignancies (PTLD, NK/T cell lymphoma) that are challenging to study or require invasive biopsies[49-52]. Thus, integrated use of fluorescent antibodies, nucleic acid probes, and functional stains alongside technological innovations (e.g., spectral detection and signal unmixing) make flow cytometry incredibly well-suited to interrogate EBV infection at single-cell resolution.

EBV heterogeneity and replication programs under the microscope: Technological improvements in microscopy have likewise vastly expanded EBV infection insights. Multicolor fluorescence microscopy demonstrated lytic cell resistance to apoptosis[53], chemotherapy-induced reactivation[54], and heterogeneous viral expression programs in B lymphoid and epithelial cells[55]. Live-cell and time-resolved acquisition captured details of EBV genome propagation in single cells through latency[56] and lytic reactivation[57,58]. In the former case, quantitative analyses demonstrated that individual EBV episomes are replicated at ~84% efficiency in S phase and are unevenly distributed to daughter cells, resulting in cell proliferative advantage by viral genome dosage[56]. This observation highlights the clinically relevant – albeit highly challenging –possibility for viral genome elimination. In lytic models, fluorescence microscopy revealed the striking formation[59,60] and evolution of intranuclear viral replication compartments (VRCs)[57,58]. Notably, VRC architecture varies with lytic program

kinetics and can be distinguished by viral genome replication status[61]. Fluorescence imaging of host DNA damage repair factors alongside lytic antigens has been instrumental in dissecting the essentiality DNA damage responses co-opted by EBV in its productive cycle[62-64]. Recently, an approach based on high-content screening and pseudotime analysis (see single-cell sequencing section) was used to recover coordinated dynamics of host DNA damage and lytic infection without live-cell acquisition[65].

Pseudobulk expression profiling and early multiomics: High-dimensional gene expression assays enabled even more detailed investigations of EBV infection dynamics in conjunction with flow cytometry and fluorescence microscopy. Time-resolved flow and mRNA microarray profiling captured proliferation and oncogenic stress dynamics correlated with latent promoter usage through primary infection and LCL outgrowth[46]. Microarray analysis of early-infected cells sorted by proliferative history demonstrated that distinct post-infection cell fates governed by metabolic stress are present within an individual timepoint[47]. LMP1-based sorting coupled to RNA-seq and FiSH analyses showed simultaneous presence of EBV programs (latency IIb and latency III) linked to B cell phenotypic heterogeneity in established LCLs[66]. Time-resolved primary infection RNA-seq studies yielded cellular and viral gene signature kinetics that further elucidated the relationship between latent programs and corresponding B cell developmental stages proposed by the GC model[67,68]. Similar to LCL datasets[69], gene regulatory mechanisms of host-virus coordination during primary infection – particularly the role of EBV super-enhancers[70] – were elaborated through multiomics integration including EBNA2, EBNA3A, and EBNA3C chromatin immunoprecipitation (ChIP) sequencing and chromatin interaction analysis with paired-end tag sequencing (ChIA-PET) data for RNA polymerase II[68]. EBNA1-activated purine metabolism was identified via temporal metabolomics alongside transcriptome profiling and assay for transposase-accessible chromatin (ATAC) sequencing[71]. Multiomics analyses of host and viral expression, regulation, and chromatin conformation have also been applied to study lytic infection and concomitant host transcriptional shutoff[72]. Bulk multiomics have also been applied to study EBV-associated PTLD[73], NK/T cell lymphoma[74], and spontaneous LCLs derived from people with multiple sclerosis (MS)[75]. While not strictly single-cell approaches, time-resolved and pseudobulk gene expression and multiomics studies clearly set the precedent for genome-wide single-cell studies of EBV-host dynamics.

II. Basic Steps in a Typical Single-Cell Sequencing Workflow

Single-cell library preparation: Individual cells are typically isolated for sequence library preparation via droplet-generating microfluidic devices (drop-seq) or sequential rounds of splitting and pooling across well plates (split-seq) (**Figure 3A**). The objective of drop-seq[76] is to optimally generate thousands of water-in-oil emulsion droplets that each encapsulate one cell and one microbead functionalized with barcoded nucleotide capture sequences in lysis buffer. RNA molecules from the cell lysed within the droplet are captured by hybridization with bead barcode sequences (most commonly, barcoded polyT capture of mRNA 3' polyA sequences or 5'-ligated universal adapters). Importantly, each barcode on a given microbead contains a common sequence identifying the cell of origin as well as a random unique molecular identifier (UMI) sequence to enable accurate transcript counting. Droplets are then broken and captured RNA molecules are reverse-transcribed (with barcodes) into cDNA molecules. The cDNA molecules are PCR-amplified and quality control (QC) analyzed for fragment length to produce libraries for sequencing. This approach is effective but is prone to artifacts (e.g., barcode swapping between beads that cross-contaminates cell reads) that can be addressed in QC steps. By comparison, split-seq[77] generates uniquely barcoded cells without the need for specialized microfluidics by employing sequential cell partitioning into well plates, barcode incorporation, and pooling. In the first plate, cDNAs with initial barcodes (one sequence per well) are produced via reverse transcription in fixed cells or nuclei (hundreds to thousands per well). Cells are pooled and randomly re-plated, after which a second barcode sequence (again, well-specific) is ligated. This process is typically repeated for three to four total barcoding rounds. For example, three barcoding rounds in 96-well plates theoretically yields 96^3 (884,736) unique barcode combinations. Generally, split-seq enables cost-effective high-throughput capture and does not require Poisson-limited cell isolation. Cells are lysed prior to the final barcode incorporation, after which cDNA pools are amplified, QC-ed, and sequenced.

Spatial technologies: Spatial-omics is a rapidly expanding technological field reviewed in depth elsewhere[78,79]. Spatial expression assays can be classified broadly into image-based or sequencing-based approaches (**Figure 3B**). Image-based assays have the advantage of single-cell (sub-cellular) resolution, whereas sequencing-based approaches trade off resolution for higher feature dimensionality (e.g., pangenome). Spatial imaging transcriptomics of dozens to hundreds of target RNAs can be achieved through iterative rounds of fluorescent probe hybridization (and, in some cases, signal amplification), image capture, and probe stripping on slide-mounted tissues or tissue arrays[80-82]. Single-cell expression maps are constructed through cell segmentation, registration, and fluorescence quantification. A similar iterative approach can be used for spatial imaging proteomics, wherein a library of sequence-barcoded antibodies are reacted with a sample and probed in rounds with fluorophore-conjugated complementary oligonucleotides (e.g., CODEX: co-detection by indexing)[83]. Sequencing-based spatial transcriptomics also utilizes target-specific oligonucleotide probes, but libraries are constructed for broader coverage with multiple probes per target. Following probe library hybridization in tissue, sequences are captured on spatially barcoded sequence arrays. This step is designed

such that synthesized cDNA libraries incorporate arrayed barcodes along with UMI and target sequences, which enables computational assignment of UMIs to spatial coordinates and tissue image registration[84].

<u>NGS and data pre-processing</u>: Library NGS, assembly, alignment, and pre-processing (**Figure 3C**) are essential for downstream analyses. Quality-controlled (QC) single-cell cDNA libraries are typically modified via index adapter ligation and sequenced on flow cells using short-read techniques (e.g., Illumina sequencing-by-synthesis). Sequencing depth is a critical consideration in single-cell experiments; appropriate flow cell capacity should be selected based on the number of recovered cells and target reads per cell. For example, paired-end sequencing of 10,000 recovered cells at a depth of 50,000 reads per cell requires 500 million read capacity – this is characteristic for a single-sample experiment. Raw NGS base calls are assembled into reads that include index, barcode/UMI, and captured target sequences.

Assembled reads are demultiplexed by sample and cell of origin and aligned (mapped) to a user-specified reference genome constructed from sequence (fasta format) and annotation (commonly in .gtf format) files. An appropriate multi-species reference genome should be tailored to the experimental design. For example, studies of EBV-infected human B cell models or clinical samples should use a reference containing human and EBV sequences (ideally, type- and strain-specific); studies of EBV-infected tumor xenografts in mice would additionally benefit from the inclusion of a murine reference.

Alignment determines read correspondence to genes and cells and produces a UMI count matrix (cell-by-feature quantification). UMI matrices are inherently sparse (zero-inflated) due to feature richness (the "curse of dimensionality") and undersampling of each cell's full transcriptome (read dropout). Increasing read depth per cell can partially reduce dropout at the expense of increased noise RNA levels. Filtering true single captured cells from ambient RNA (e.g., empty drops) and cell multiplets is a key pre-processing QC step and is typically achieved with statistical methods[85,86] but may require tuning based on system-specific biology. Additional QC filters can be applied to UMI data to remove cells with uncharacteristically low or high feature diversity (unique genes), total reads, or mitochondrial read content, among other parameters. Specific QC threshold values should be tailored by factors including read depth and sample biology. For example, historically recommended mitochondrial fraction thresholds (often <5% of reads) used to filter dead or dying cells were informed by early analyses of resting PBMCs; cell lines and tumor tissue regularly exceed such levels due to biologically meaningful activities[87,88].

Following QC, expression data are normalized to account for PCR amplification biases and differential read depth across individual cells. Features with high-variance intercellular expression are identified, and normalized expression data are scaled to avoid overweighting high-variance features. Optionally, specific genes can be regressed out during the scaling process to remove their contribution to downstream dimensional reduction and clustering. Dimensional reduction is generally performed in two phases. First, principal component analysis (PCA) is used to summarize the variation across tens of thousands of features (genes) into PCA dimensions (usually between 10 to 50 components) that maximally account for genome-wide cell variance. Second, PCs are further projected into two-dimensional (or occasionally, 3D) space via uniform manifold

approximation projection (UMAP)[89] or t-distributed stochastic neighbor embedding (t-SNE)[90] techniques. After completion of these steps, numerous analytical techniques can be applied to discover and compare cell phenotypes and biological activities.

Single-cell data analysis: Powerful open-source software suites have been developed for single-cell data analysis[91-93]. Across platforms, grouping of dimensionally reduced datapoints by expression similarity ("clustering") is a key early step in unsupervised phenotyping (**Figure 3D**). This clustering is best achieved by statistical methods including calculation of each cell's k-nearest neighbors (K-NN)[94,95] and community classification techniques (e.g., Louvain or Leiden algorithms)[96,97]. The number and granularity of resulting clusters can be tuned via resolution parameters to avoid under- or over-fitting. Dataset-optimized resolution parameters can be calculated, though some degree of manual tuning may be appropriate for specific biological samples. Whereas clustering *per se* may be relatively straightforward, biological annotation and interpretation of clusters is a perennial sample-specific challenge. Several bioinformatic tools have been developed to assign cell types to clusters and spatial capture spots based on pre-existing or user-curated expression signatures[98-100]. However, these expression profiles are generally derived from "normal" cell states; proper annotation of EBV-infected cells and tissues often demands additional considerations including *a priori* biological knowledge.

Sequencing depth may be sufficient to resolve biological phenotypes but nonetheless inadequate to capture important biological differences due to transcript dropout. This technical limitation particularly affects low-abundance targets including many transcription factors. An optional read imputation step may be performed to mitigate technical false negatives due to dropout (**Figure 3E**). The overall objective of this step should be to recover biologically "true" non-zeros without introducing artifactual false positives for unexpressed genes. Many statistical approaches have been implemented to address dropout, each with relative performance advantages and disadvantages[101]. Ultimately, imputed count matrices should be cross-referenced against original read counts and validated by orthogonal assays when possible.

Cluster-level transcriptomes can be compared through differential expression (DE) analyses to define biological phenotype markers. Beyond "shiny pebble" exploration, ranked DE gene lists for clusters of interest can be analyzed with ontology and enrichment tools to recover significantly upregulated processes or cell responses[102-107] (**Figure 3F**). Cluster-level gene enrichment can be used to infer activities of known cell signaling pathways and specific transcription factors. Several bioinformatic tools are available to conduct such gene regulatory network (GRN) analysis[108-110] (**Figure 3G**). An in-depth review of GRN techniques for single-cell data is available for interested readers[111]. As in cluster annotation, DE enrichment and GRN results should be interpreted in the context of sample-specific knowledge including viral expression signatures unaccounted by human-only databases.

Advances in single-cell library strategies now accommodate direct multiomics measurements from individual cells, which bolster gene regulatory predictions beyond inference from expression alone. In many cases, gene expression (scRNA-seq) can be co-assayed with chromatin accessibility (scATAC-seq), cell surface proteins (CITE-seq), and/or immunoreceptor specificity (BCR/TCR; scVDJ-seq). Analytically, digesting different

molecular information layers depends on correlating discovered phenotypes across assays. Transfer anchor integration[93,112,113] is an effective approach to accomplish multimodal data integration (**Figure 3H**). Fundamentally, transfer integration leverages pairwise single-cell similarities across each assay to define a common multimodal phenotypic space[112]. Single-cell or pseudobulk resolution obtained in one or more modalities may also be conferred to population ensemble measurements from another modality, as has been demonstrated for scRNA-seq, scATAC-seq and bulk ChIP-seq data from EBV-infected B cells[114].

The existence of distinct biologic states in samples of a purported cell type (e.g., EBV-infected B cells) found by single-cell methods raises an important question: how are phenotypic states related? Pseudotime and velocity methods[115-118] (**Figure 3I**) originally demonstrated for resolving cell fate decisions in mammalian development are well-suited to address this question. These methods use graph-based analysis of transcriptomic similarity to infer biological state transitions within and across static timepoints. Thus, samples that contain heterogeneous mixtures of dynamic biologic states may be used to construct coherent biological states and response trajectories. For EBV models, such trajectories effectively provide reaction coordinates for the progress or stage of infection.

A second question is raised by the presence of heterogeneous cell types in experimental models and particularly in clinical samples: how might different cell types interact? Cell-cell communication inference tools designed for single-cell and spatial transcriptomics data[119,120] can be applied to make such predictions (**Figure 3J**). These approaches infer intra- and inter-phenotype ligand-receptor interactions encompassing direct cell-to-cell contact and signaling via secreted factors. As for gene enrichment and GRN predictions, the databases used by cell-cell communication software are limited to known protein interactions and do not include EBV genes. While inference from the mRNA level does not guarantee the occurrence of protein-protein interactions, these bioinformatic tools can nonetheless identify important axes of oncogenesis and immune response.

Spatial datasets capture biological dimensions that present additional analytic challenges and opportunities. Numerous methodological approaches have been developed to study gene expression using *in situ* context[121-126]. The precise solution for this task is typically platform-dependent; however, the underlying goal is to resolve biological phenotypes and interactions within global and local tissue neighborhoods (**Figure 3K**). One approach measures expression gradients along user-defined trajectories[126], while another class of methods calculates spatially differential expression patterns[121]. A separate direction-agnostic approach to map expression gradients relative to annotated tissue types of interest has been demonstrated recently in a study of EBV-associated HIV-NHL[127]. In principle, spatial adjacency may be used to constrain or otherwise weight cell-cell communication interactions and contextualize biological activity enrichment. Thus, comprehensive single-cell and spatial analysis workflows characteristically require implementation of multiple bioinformatic methods in concert.

SUPPLEMENTARY REFERENCES

1 Miller Mary, H., Stitt, D. & Miller, G. Epstein-Barr Viral Antigen in Single Cell Clones of Two Human Leukocytic Lines. *Journal of Virology* **6**, 699-701 (1970). https://doi.org/10.1128/jvi.6.5.699-701.1970

2 Sugden, B. & Mark, W. Clonal transformation of adult human leukocytes by Epstein-Barr virus. *Journal of Virology* **23**, 503-508 (1977). https://doi.org/doi:10.1128/jvi.23.3.503-508.1977

3 Sugden, B., Phelps, M. & Domoradzki, J. Epstein-Barr virus DNA is amplified in transformed lymphocytes. *Journal of Virology* **31**, 590-595 (1979). https://doi.org/doi:10.1128/jvi.31.3.590-595.1979

4 Kirchmaier, A. L. & Sugden, B. Plasmid maintenance of derivatives of oriP of Epstein-Barr virus. *Journal of Virology* **69**, 1280-1283 (1995). https://doi.org/doi:10.1128/jvi.69.2.1280-1283.1995

5 Shimizu, N., Tanabe-Tochikura, A., Kuroiwa, Y. & Takada, K. Isolation of Epstein-Barr virus (EBV)-negative cell clones from the EBV-positive Burkitt's lymphoma (BL) line Akata: malignant phenotypes of BL cells are dependent on EBV. *Journal of virology* **68**, 6069-6073 (1994).

6 Shimizu, N., Yoshiyama, H. & Takada, K. Clonal propagation of Epstein-Barr virus (EBV) recombinants in EBV-negative Akata cells. *Journal of virology* **70**, 7260-7263 (1996).

7 Lindahl, T. *et al.* Covalently closed circular duplex DNA of Epstein-Barr virus in a human lymphoid cell line. *Journal of Molecular Biology* **102**, 511-530 (1976). https://doi.org/https://doi.org/10.1016/0022-2836(76)90331-4

8 Raab-Traub, N. & Flynn, K. The structure of the termini of the Epstein-Barr virus as a marker of clonal cellular proliferation. *Cell* **47**, 883-889 (1986). https://doi.org/https://doi.org/10.1016/0092-8674(86)90803-2

9 Moody, C. A., Scott, R. S., Su, T. & Sixbey, J. W. Length of Epstein-Barr virus termini as a determinant of epithelial cell clonal emergence. *Journal of virology* **77**, 8555-8561 (2003).

10 Riel, M. & Johannsen, E. C. in *Current Topics in Microbiology and Immunology* 1-37 (Springer Berlin Heidelberg, 2025).

11 Cleary, M. L., Nalesnik, M. A., Shearer, W. T. & Sklar, J. Clonal analysis of transplant-associated lymphoproliferations based on the structure of the genomic termini of the Epstein-Barr virus. *Blood* **72** (1988).

12 Knecht, H. *et al.* Detection of Epstein-Barr virus DNA by polymerase chain reaction in lymph node biopsies from patients with angioimmunoblastic lymphadenopathy. *British journal of haematology* **75**, 610-614 (1990).

13 Teramoto, N., Akagi, T., Yoshino, T., Takahashi, K. & Jeon, H. J. Direct detection of Epstein-Barr virus DNA from a single Reed-Sternberg cell of Hodgkin's disease by polymerase chain reaction. *Jpn J Cancer Res* **83**, 329-333 (1992). https://doi.org/10.1111/j.1349-7006.1992.tb00110.x

14 Roth, J., Daus, H., Gause, A., Trümper, L. & Pfreundschuh, M. Detection of Epstein-Barr Virus DNA in Hodgkin- and Reed-Sternberg-Cells by Single Cell PCR. *Leukemia & Lymphoma* **13**, 137-142 (1994). https://doi.org/10.3109/10428199409051664

15 Leeuwenhoek, A. v. & De Graaf, R. A specimen of some observations made by a microscope, contrived by M. Leewenhoeck in Holland, lately communicated by Dr. Regnerus de Graaf. *Philosophical Transactions of the Royal Society of London* **8**, 6037-6038 (1673).

16 Leeuwenhoek, A. v. 1677. Letter of October 9, 1676 to the Royal Society. *Philosophical Transactions of the Royal Society of London* **12**, 821-831 (1676).

17 Miller, G., Shope, T., Lisco, H., Stitt, D. & Lipman, M. Epstein-Barr Virus: Transformation, Cytopathic Changes, and Viral Antigens in Squirrel Monkey and Marmoset Leukocytes. *Proceedings of the National Academy of Sciences* **69**, 383-387 (1972). https://doi.org/doi:10.1073/pnas.69.2.383

18 Menezes, J., Leibold, W. & Klein, G. Biological differences between epstein-barr virus (EBV) strains with regard to lymphocyte transforming ability, superinfection and antigen induction. *Experimental Cell Research* **92**, 478-484 (1975). https://doi.org/https://doi.org/10.1016/0014-4827(75)90404-8

19 Klein, G. *et al.* Direct Evidence for the Presence of Epstein-Barr Virus DNA and Nuclear Antigen in Malignant Epithelial Cells from Patients with Poorly Differentiated Carcinoma of the Nasopharynx. *Proceedings of the National Academy of Sciences* **71**, 4737-4741 (1974). https://doi.org/doi:10.1073/pnas.71.12.4737

20 Anagnostopoulos, I. *et al.* Heterogeneous Epstein-Barr virus infection patterns in peripheral T- cell lymphoma of angioimmunoblastic lymphadenopathy type. *Blood* **80**, 1804-1812 (1992). https://doi.org/10.1182/blood.V80.7.1804.1804

21 Brink, A. A. *et al.* Presence of Epstein-Barr virus latency type III at the single cell level in post-transplantation lymphoproliferative disorders and AIDS related lymphomas. *Journal of Clinical Pathology* **50**, 911 (1997). https://doi.org/10.1136/jcp.50.11.911

22 Hänel, P., Hummel, M., Anagnostopoulos, I. & Stein, H. Analysis of single EBER-positive and negative tumour cells in EBV-harbouring B-cell non-Hodgkin lymphomas. *The Journal of Pathology* **195**, 355-360 (2001). https://doi.org/https://doi.org/10.1002/path.954

23 Niedobitek, G. *et al.* Epstein-Barr virus (EBV) infection in infectious mononucleosis: virus latency, replication and phenotype of EBV-infected cells. *The Journal of pathology* **182**, 151-159 (1997).

24 Gregory, C., Rowe, M. & Rickinson, A. Different Epstein-Barr virus-B cell interactions in phenotypically distinct clones of a Burkitt's lymphoma cell line. *Journal of General Virology* **71**, 1481-1495 (1990).

25 Kelly, G. L., Milner, A. E., Baldwin, G. S., Bell, A. I. & Rickinson, A. B. Three restricted forms of Epstein–Barr virus latency counteracting apoptosis in c-myc-expressing Burkitt lymphoma cells. *Proceedings of the National Academy of Sciences* **103**, 14935-14940 (2006).

26 Rowe, M. *et al.* Epstein-Barr virus (EBV)-associated lymphoproliferative disease in the SCID mouse model: implications for the pathogenesis of EBV-positive lymphomas in man. *Journal of Experimental Medicine* **173**, 147-158 (1991). https://doi.org/10.1084/jem.173.1.147

27 Oyama, T. *et al.* Senile EBV+ B-cell lymphoproliferative disorders: a clinicopathologic study of 22 patients. *The American journal of surgical pathology* **27**, 16-26 (2003).

28 Yarchoan, R., Tosato, G., Blaese, R., Simon, R. & Nelson, D. Limiting dilution analysis of Epstein-Barr virus-induced immunoglobulin production by human B cells. *The Journal of experimental medicine* **157**, 1-14 (1983).

29 Thorley-Lawson, D. A. & Mann, K. P. Early events in Epstein-Barr virus infection provide a model for B cell activation. *The Journal of experimental medicine* **162**, 45-59 (1985).

30 Roughan Jill, E. & Thorley-Lawson David, A. The Intersection of Epstein-Barr Virus with the Germinal Center. *Journal of Virology* **83**, 3968-3976 (2009). https://doi.org/10.1128/jvi.02609-08

31 Torgbor, C., Thorley-Lawson, D. A. & Moormann, A. M. Epstein-Barr virus-infected tonsillar marginal zone B cells <i>in vivo</i> as a precursor for immunosuppression-related B-cell lymphoma. *Journal of Virology* **99**, e01051-01024 (2025). https://doi.org/doi:10.1128/jvi.01051-24

32 Mancao, C., Altmann, M., Jungnickel, B. & Hammerschmidt, W. Rescue of "crippled" germinal center B cells from apoptosis by Epstein-Barr virus. *Blood* **106**, 4339-4344 (2005). https://doi.org/10.1182/blood-2005-06-2341

33 Tracy Sean, I. *et al.* Persistence of Epstein-Barr Virus in Self-Reactive Memory B Cells. *Journal of Virology* **86**, 12330-12340 (2012). https://doi.org/10.1128/jvi.01699-12

34 Hadinoto, V., Shapiro, M., Sun, C. C. & Thorley-Lawson, D. A. The Dynamics of EBV Shedding Implicate a Central Role for Epithelial Cells in Amplifying Viral Output. *PLOS Pathogens* **5**, e1000496 (2009). https://doi.org/10.1371/journal.ppat.1000496

35 Miyashita, E. M., Yang, B., Babcock, G. J. & Thorley-Lawson, D. A. Identification of the site of Epstein-Barr virus persistence in vivo as a resting B cell. *Journal of Virology* **71**, 4882-4891 (1997). https://doi.org/doi:10.1128/jvi.71.7.4882-4891.1997

36 Babcock, G. J., Decker, L. L., Volk, M. & Thorley-Lawson, D. A. EBV persistence in memory B cells in vivo. *Immunity* **9**, 395-404 (1998).

37 Babcock, G. J., Hochberg, D. & Thorley-Lawson, D. A. The Expression Pattern of Epstein-Barr Virus Latent Genes In Vivo Is Dependent upon the Differentiation Stage of the Infected B Cell. *Immunity* **13**, 497-506 (2000). https://doi.org/10.1016/S1074-7613(00)00049-2

38 Babcock, G. J. & Thorley-Lawson, D. A. Tonsillar memory B cells, latently infected with Epstein–Barr virus, express the restricted pattern of latent genes previously found only in Epstein–Barr virus-associated tumors. *Proceedings of the National Academy of Sciences* **97**, 12250-12255 (2000). https://doi.org/doi:10.1073/pnas.200366597

39 Laichalk, L. L. & Thorley-Lawson, D. A. Terminal differentiation into plasma cells initiates the replicative cycle of Epstein-Barr virus in vivo. *Journal of virology* **79**, 1296-1307 (2005).

40 Rodriguez, A., Jung, E. J. & Flemington, E. K. Cell Cycle Analysis of Epstein-Barr Virus-Infected Cells following Treatment with Lytic Cycle-Inducing Agents. *Journal of Virology* **75**, 4482-4489 (2001). https://doi.org/doi:10.1128/jvi.75.10.4482-4489.2001

41 Allday, M., Inman, G. J., Crawford, D. H. & Farrell, P. J. DNA damage in human B cells can induce apoptosis, proceeding from G1/S when p53 is transactivation competent and G2/M when it is transactivation defective. *The EMBO journal* **14**, 4994-5005 (1995).

42 Parker, G. A., Touitou, R. & Allday, M. J. Epstein-Barr virus EBNA3C can disrupt multiple cell cycle checkpoints and induce nuclear division divorced from cytokinesis. *Oncogene* **19**, 700-709 (2000).

43 Wade, M. & Allday, M. J. Epstein-Barr virus suppresses a G2/M checkpoint activated by genotoxins. *Molecular and cellular biology* **20**, 1344-1360 (2000).

44 Leao, M., Anderton, E., Wade, M., Meekings, K. & Allday, M. J. Epstein-barr virus-induced resistance to drugs that activate the mitotic spindle assembly checkpoint in Burkitt's lymphoma cells. *Journal of virology* **81**, 248-260 (2007).

45 Shannon-Lowe, C. *et al.* Epstein–Barr virus-induced B-cell transformation: quantitating events from virus binding to cell outgrowth. *Journal of General Virology* **86**, 3009-3019 (2005). https://doi.org/https://doi.org/10.1099/vir.0.81153-0

46 Nikitin, P. A. *et al.* An ATM/Chk2-mediated DNA damage-responsive signaling pathway suppresses Epstein-Barr virus transformation of primary human B cells. *Cell host & microbe* **8**, 510-522 (2010).

47 McFadden, K. *et al.* Metabolic stress is a barrier to Epstein–Barr virus-mediated B-cell immortalization. *Proceedings of the National Academy of Sciences* **113**, E782-E790 (2016).

48 Willard, K. A. *et al.* Viral and host factors drive a type 1 Epstein–Barr virus spontaneous lytic phenotype. *Mbio* **14**, e02204-02223 (2023).

49 Kimura, H. *et al.* Identification of Epstein-Barr virus (EBV)–infected lymphocyte subtypes by flow cytometric in situ hybridization in EBV-associated lymphoproliferative diseases. *The Journal of infectious diseases* **200**, 1078-1087 (2009).

50 Kawabe, S. *et al.* Application of flow cytometric in situ hybridization assay to E pstein–B arr virus-associated T/natural killer cell lymphoproliferative diseases. *Cancer science* **103**, 1481-1488 (2012).

51 Fournier, B. *et al.* Rapid identification and characterization of infected cells in blood during chronic active Epstein-Barr virus infection. *Journal of Experimental Medicine* **217** (2020). https://doi.org/10.1084/jem.20192262

52 Tomomasa, D. *et al.* Highly sensitive detection of Epstein-Barr virus-infected cells by EBER flow FISH. *International Journal of Hematology* **120**, 241-251 (2024). https://doi.org/10.1007/s12185-024-03786-0

53 Inman, G. J., Binné, U. K., Parker, G. A., Farrell, P. J. & Allday, M. J. Activators of the Epstein-Barr virus lytic program concomitantly induce apoptosis, but lytic gene expression protects from cell death. *Journal of virology* **75**, 2400-2410 (2001).

54 Feng, W.-h., Hong, G., Delecluse, H.-J. & Kenney, S. C. Lytic induction therapy for Epstein-Barr virus-positive B-cell lymphomas. *Journal of virology* **78**, 1893-1902 (2004).

55 Shannon-Lowe, C. *et al.* Features Distinguishing Epstein-Barr Virus Infections of Epithelial Cells and B Cells: Viral Genome Expression, Genome Maintenance, and Genome Amplification. *Journal of Virology* **83**, 7749-7760 (2009). https://doi.org/10.1128/jvi.00108-09

56 Nanbo, A., Sugden, A. & Sugden, B. The coupling of synthesis and partitioning of EBV's plasmid replicon is revealed in live cells. *The EMBO Journal* **26**, 4252-4262 (2007). https://doi.org/https://doi.org/10.1038/sj.emboj.7601853

57 Chiu, Y.-F., Sugden, A. U. & Sugden, B. Epstein-Barr viral productive amplification reprograms nuclear architecture, DNA replication, and histone deposition. *Cell host & microbe* **14**, 607-618 (2013).

58 Nagaraju, T., Sugden, A. U. & Sugden, B. Four-dimensional analyses show that replication compartments are clonal factories in which Epstein–Barr viral DNA amplification is coordinated. *Proceedings of the National Academy of Sciences* **116**, 24630-24638 (2019).

59 Bell, P., Lieberman, P. M. & Maul, G. G. Lytic but not latent replication of Epstein-Barr virus is associated with PML and induces sequential release of nuclear domain 10 proteins. *Journal of virology* **74**, 11800-11810 (2000).

60 Daikoku, T. *et al.* Architecture of replication compartments formed during Epstein-Barr virus lytic replication. *Journal of virology* **79**, 3409-3418 (2005).

61 Rosemarie, Q., Kirschstein, E. & Sugden, B. How Epstein-Barr Virus induces the reorganization of cellular chromatin. *MBio* **14**, e02686-02622 (2023).

62 Hau, P. M. *et al.* Role of ATM in the formation of the replication compartment during lytic replication of Epstein-Barr virus in nasopharyngeal epithelial cells. *Journal of virology* **89**, 652-668 (2015).

63 Wang'Ondu, R. *et al.* DNA damage signaling is induced in the absence of Epstein—Barr virus (EBV) lytic DNA replication and in response to expression of ZEBRA. *PLoS One* **10**, e0126088 (2015).

64 Salamun, S. G. *et al.* The Epstein-Barr virus BMRF1 protein activates transcription and inhibits the DNA damage response by binding NuRD. *Journal of Virology* **93**, 10.1128/jvi. 01070-01019 (2019).

65 Tekle, D. G., Sexton, J. Z. & SoRelle, E. D. Reconstructing EBV reactivation and DNA damage response kinetics in morphologic pseudotime. *BioRxiv* (2025). https://doi.org/https://doi.org/10.1101/2025.10.06.680675

66 Messinger Joshua, E., Dai, J., Stanland Lyla, J., Price Alexander, M. & Luftig Micah, A. Identification of Host Biomarkers of Epstein-Barr Virus Latency IIb and Latency III. *mBio* **10**, 10.1128/mbio.01006-01019 (2019). https://doi.org/10.1128/mbio.01006-19

67 Mrozek-Gorska, P. *et al.* Epstein–Barr virus reprograms human B lymphocytes immediately in the prelatent phase of infection. *Proceedings of the National Academy of Sciences* **116**, 16046-16055 (2019). https://doi.org/doi:10.1073/pnas.1901314116

68 Wang, C. *et al.* RNA Sequencing Analyses of Gene Expression during Epstein-Barr Virus Infection of Primary B Lymphocytes. *J Virol* **93** (2019). https://doi.org/10.1128/jvi.00226-19

69 Arvey, A. *et al.* An atlas of the Epstein-Barr virus transcriptome and epigenome reveals host-virus regulatory interactions. *Cell host & microbe* **12**, 233-245 (2012).

70 Zhou, H. *et al.* Epstein-Barr virus oncoprotein super-enhancers control B cell growth. *Cell Host Microbe* **17**, 205-216 (2015). https://doi.org/10.1016/j.chom.2014.12.013

71 Lamontagne, R. J. *et al.* A multi-omics approach to Epstein-Barr virus immortalization of B-cells reveals EBNA1 chromatin pioneering activities targeting nucleotide metabolism. *PLoS Pathogens* **17**, e1009208 (2021).

72 Buschle, A. *et al.* Epstein-Barr virus inactivates the transcriptome and disrupts the chromatin architecture of its host cell in the first phase of lytic reactivation. *Nucleic acids research* **49**, 3217-3241 (2021).

73 Toh, J. *et al.* Multi-modal analysis reveals tumor and immune features distinguishing EBV-positive and EBV-negative post-transplant lymphoproliferative disorders. *Cell Reports Medicine* **5** (2024). https://doi.org/10.1016/j.xcrm.2024.101851

74 Akazawa, R. *et al.* Multiomics analysis reveals the genetic and epigenetic features of high-risk NK cell-type chronic active EBV infection. *Blood* (2025). https://doi.org/10.1182/blood.2024026805

75 Soldan, S. S. *et al.* Multiple sclerosis patient-derived spontaneous B cells have distinct EBV and host gene expression profiles in active disease. *Nature Microbiology* **9**, 1540-1554 (2024). https://doi.org/10.1038/s41564-024-01699-6

76 Macosko, Evan Z. *et al.* Highly Parallel Genome-wide Expression Profiling of Individual Cells Using Nanoliter Droplets. *Cell* **161**, 1202-1214 (2015). https://doi.org/10.1016/j.cell.2015.05.002

77 Rosenberg, A. B. *et al.* Single-cell profiling of the developing mouse brain and spinal cord with split-pool barcoding. *Science* **360**, 176-182 (2018). https://doi.org/doi:10.1126/science.aam8999

78 Tian, L., Chen, F. & Macosko, E. Z. The expanding vistas of spatial transcriptomics. *Nature Biotechnology* **41**, 773-782 (2023). https://doi.org/10.1038/s41587-022-01448-2

79 Liu, L. *et al.* Spatiotemporal omics for biology and medicine. *Cell* **187**, 4488-4519 (2024). https://doi.org/10.1016/j.cell.2024.07.040

80 Ke, R. *et al.* In situ sequencing for RNA analysis in preserved tissue and cells. *Nature methods* **10**, 857-860 (2013).

81 Lee, J. H. *et al.* Highly multiplexed subcellular RNA sequencing in situ. *science* **343**, 1360-1363 (2014).

82 Chen, K. H., Boettiger, A. N., Moffitt, J. R., Wang, S. & Zhuang, X. Spatially resolved, highly multiplexed RNA profiling in single cells. *Science* **348**, aaa6090 (2015).

83 Goltsev, Y. *et al.* Deep Profiling of Mouse Splenic Architecture with CODEX Multiplexed Imaging. *Cell* **174**, 968-981.e915 (2018). https://doi.org/10.1016/j.cell.2018.07.010

84 Oliveira, M. F. *et al.* Characterization of immune cell populations in the tumor microenvironment of colorectal cancer using high definition spatial profiling. *BioRxiv*, 2024.2006. 2004.597233 (2024).

85 Lun, A. T. *et al.* EmptyDrops: distinguishing cells from empty droplets in droplet-based single-cell RNA sequencing data. *Genome biology* **20**, 63 (2019).

86 McGinnis, C. S., Murrow, L. M. & Gartner, Z. J. DoubletFinder: doublet detection in single-cell RNA sequencing data using artificial nearest neighbors. *Cell systems* **8**, 329-337. e324 (2019).

87 Osorio, D. & Cai, J. J. Systematic determination of the mitochondrial proportion in human and mice tissues for single-cell RNA-sequencing data quality control. *Bioinformatics* **37**, 963-967 (2020). https://doi.org/10.1093/bioinformatics/btaa751

88 Yates, J., Kraft, A. & Boeva, V. Filtering cells with high mitochondrial content depletes viable metabolically altered malignant cell populations in cancer single-cell studies. *Genome Biology* **26**, 91 (2025). https://doi.org/10.1186/s13059-025-03559-w

89 McInnes, L., Healy, J. & Melville, J. Umap: Uniform manifold approximation and projection for dimension reduction. *arXiv preprint arXiv:1802.03426* (2018).

90 Maaten, L. v. d. & Hinton, G. Visualizing data using t-SNE. *Journal of machine learning research* **9**, 2579-2605 (2008).

91 Satija, R., Farrell, J. A., Gennert, D., Schier, A. F. & Regev, A. Spatial reconstruction of single-cell gene expression data. *Nature Biotechnology* **33**, 495-502 (2015). https://doi.org/10.1038/nbt.3192

92 Wolf, F. A., Angerer, P. & Theis, F. J. SCANPY: large-scale single-cell gene expression data analysis. *Genome Biology* **19**, 15 (2018). https://doi.org/10.1186/s13059-017-1382-0

93 Hao, Y. *et al.* Dictionary learning for integrative, multimodal and scalable single-cell analysis. *Nature biotechnology* **42**, 293-304 (2024).

94 Fix, E. & Hodges, J. L. *Discriminatory analysis: nonparametric discrimination, consistency properties*. Vol. 1 (USAF school of Aviation Medicine, 1985).

95 Cover, T. & Hart, P. Nearest neighbor pattern classification. *IEEE transactions on information theory* **13**, 21-27 (1967).

96 Blondel, V. D., Guillaume, J.-L., Lambiotte, R. & Lefebvre, E. Fast unfolding of communities in large networks. *Journal of statistical mechanics: theory and experiment* **2008**, P10008 (2008).

97 Traag, V. A., Waltman, L. & Van Eck, N. J. From Louvain to Leiden: guaranteeing well-connected communities. *Scientific reports* **9**, 1-12 (2019).

98 Hao, Y. *et al.* Integrated analysis of multimodal single-cell data. *Cell* **184**, 3573-3587.e3529 (2021). https://doi.org/10.1016/j.cell.2021.04.048

99 Ianevski, A., Giri, A. K. & Aittokallio, T. Fully-automated and ultra-fast cell-type identification using specific marker combinations from single-cell transcriptomic data. *Nature Communications* **13**, 1246 (2022). https://doi.org/10.1038/s41467-022-28803-w

100 Elosua-Bayes, M., Nieto, P., Mereu, E., Gut, I. & Heyn, H. SPOTlight: seeded NMF regression to deconvolute spatial transcriptomics spots with single-cell transcriptomes. *Nucleic acids research* **49**, e50-e50 (2021).

101 Hou, W., Ji, Z., Ji, H. & Hicks, S. C. A systematic evaluation of single-cell RNA-sequencing imputation methods. *Genome biology* **21**, 218 (2020).

102 Ashburner, M. *et al.* Gene ontology: tool for the unification of biology. *Nature genetics* **25**, 25-29 (2000).

103 Kanehisa, M. & Goto, S. KEGG: kyoto encyclopedia of genes and genomes. *Nucleic acids research* **28**, 27-30 (2000).

104 Kanehisa, M., Furumichi, M., Tanabe, M., Sato, Y. & Morishima, K. KEGG: new perspectives on genomes, pathways, diseases and drugs. *Nucleic acids research* **45**, D353-D361 (2017).

105 Subramanian, A. *et al.* Gene set enrichment analysis: a knowledge-based approach for interpreting genome-wide expression profiles. *Proceedings of the National Academy of Sciences* **102**, 15545-15550 (2005).

106 Wu, T. *et al.* clusterProfiler 4.0: A universal enrichment tool for interpreting omics data. *The innovation* **2** (2021).

107 Aleksander, S. A. *et al.* The gene ontology knowledgebase in 2023. *Genetics* **224**, iyad031 (2023).

108 Holland, C. H., Szalai, B. & Saez-Rodriguez, J. Transfer of regulatory knowledge from human to mouse for functional genomics analysis. *Biochimica et Biophysica Acta (BBA) - Gene Regulatory Mechanisms* **1863**, 194431 (2020). https://doi.org/https://doi.org/10.1016/j.bbagrm.2019.194431

109 Holland, C. H. *et al.* Robustness and applicability of transcription factor and pathway analysis tools on single-cell RNA-seq data. *Genome Biology* **21**, 36 (2020). https://doi.org/10.1186/s13059-020-1949-z

110 Badia-i-Mompel, P. *et al.* decoupleR: ensemble of computational methods to infer biological activities from omics data. *Bioinformatics Advances* **2** (2022). https://doi.org/10.1093/bioadv/vbac016

111 Badia-i-Mompel, P. *et al.* Gene regulatory network inference in the era of single-cell multi-omics. *Nature Reviews Genetics* **24**, 739-754 (2023). https://doi.org/10.1038/s41576-023-00618-5

112 Stuart, T. *et al.* Comprehensive integration of single-cell data. *cell* **177**, 1888-1902. e1821 (2019).

113 Stuart, T., Srivastava, A., Madad, S., Lareau, C. A. & Satija, R. Single-cell chromatin state analysis with Signac. *Nature Methods* **18**, 1333-1341 (2021). https://doi.org/10.1038/s41592-021-01282-5

114 SoRelle, E. D. *et al.* Epstein-Barr virus evades restrictive host chromatin closure by subverting B cell activation and germinal center regulatory loci. *Cell reports* **42** (2023).

115 Trapnell, C. *et al.* The dynamics and regulators of cell fate decisions are revealed by pseudotemporal ordering of single cells. *Nature biotechnology* **32**, 381-386 (2014).

116 Cao, J. *et al.* The single-cell transcriptional landscape of mammalian organogenesis. *Nature* **566**, 496-502 (2019).

117 La Manno, G. *et al.* RNA velocity of single cells. *Nature* **560**, 494-498 (2018).

118 Bergen, V., Lange, M., Peidli, S., Wolf, F. A. & Theis, F. J. Generalizing RNA velocity to transient cell states through dynamical modeling. *Nature biotechnology* **38**, 1408-1414 (2020).

119 Efremova, M., Vento-Tormo, M., Teichmann, S. A. & Vento-Tormo, R. CellPhoneDB: inferring cell–cell communication from combined expression of multi-subunit ligand–receptor complexes. *Nature protocols* **15**, 1484-1506 (2020).

120 Jin, S. *et al.* Inference and analysis of cell-cell communication using CellChat. *Nature communications* **12**, 1088 (2021).

121 Svensson, V., Teichmann, S. A. & Stegle, O. SpatialDE: identification of spatially variable genes. *Nature Methods* **15**, 343-346 (2018). https://doi.org/10.1038/nmeth.4636

122 Zhao, E. *et al.* Spatial transcriptomics at subspot resolution with BayesSpace. *Nature biotechnology* **39**, 1375-1384 (2021).

123 Dries, R. *et al.* Giotto: a toolbox for integrative analysis and visualization of spatial expression data. *Genome biology* **22**, 78 (2021).

124 Luca, B. A. *et al.* Atlas of clinically distinct cell states and ecosystems across human solid tumors. *Cell* **184**, 5482-5496.e5428 (2021). https://doi.org/https://doi.org/10.1016/j.cell.2021.09.014

125 Steen, C. B. *et al.* The landscape of tumor cell states and ecosystems in diffuse large B cell lymphoma. *Cancer Cell* **39**, 1422-1437.e1410 (2021). https://doi.org/10.1016/j.ccell.2021.08.011

126 Ravi, V. M. *et al.* Spatially resolved multi-omics deciphers bidirectional tumor-host interdependence in glioblastoma. *Cancer cell* **40**, 639-655. e613 (2022).

127 Chadburn, A. *et al.* HIV-associated non-Hodgkin lymphoma tumor-microenvironment axes differ by EBV status across cellular origins. *bioRxiv*, 2025.2010.2015.682084 (2025). https://doi.org/10.1101/2025.10.15.682084
